# Trends in hospital admissions for childhood fractures in England

**DOI:** 10.1136/bmjpo-2021-001187

**Published:** 2021-11-09

**Authors:** Ben Arthur Marson, Joseph C Manning, Marilyn James, Adeel Ikram, David J Bryson, Benjamin J Ollivere

**Affiliations:** 1 Orthopaedics and Trauma, University of Nottingham School of Medicine, Nottingham, UK; 2 Nottingham Children’s Hospital, Nottingham University Hospitals NHS Trust, Nottingham, UK; 3 Children and Young People Health Research, School of Health Sciences, University of Nottingham, Nottingham, UK; 4 Clinical Trials Unit, University of Nottingham School of Medicine, Nottingham, UK

**Keywords:** epidemiology

## Abstract

**Purpose:**

Fractures to the axial and appendicular skeleton are common in children causing loss of opportunities and disability. There are relatively few studies available to quantify the number of children who have their fractures diagnosed in the emergency department and are then admitted to hospital for ongoing management. The purpose of this study is to explore trends of frequency, types and age of children sustaining fractures who were admitted for intervention to National Health Service (NHS) hospitals.

**Design:**

The study uses data from the Hospital Episode Statistics and Office for National Statistics from 2012 to 2019 to calculate the annual incidence of hospital admission for limb, spine, facial and skull fractures per 100 000 children.

**Results:**

During 2012–2019, 368 120 children were admitted to English NHS hospitals with a fracture. 256 008 (69.5%) were upper limb fractures, 85 737 (23.3%) were lower limb fractures and 20 939 (5.7%) were skull or facial fractures. The annual incidence of upper limb fractures was highest in children aged 5–9 (348.3 per 100 000 children) and the highest incidence of lower limb fractures was in children aged 10–15 (126.5 per 100 000 children). The incidence of skull and facial fractures in preschool (age 0–4) children has been increasing at a rate of 0.629 per 100 000 children per year.

**Implications:**

The annual incidence of hospital admission for fractures in children has been shown to be consistent for several fracture types between 2012 and 2019. An increasing trend of admissions with preschool skull fractures was observed, though the study data do not have sufficient granularity to demonstrate if this is due to changes in practice or to accidental or non-accidental causes.

**What is known about the subject?**

- Fractures in children are common with more hospital admissions for upper limb than lower limb fractures.
- Previous estimates of hospital admissions following childhood fractures are limited by a lack of robust denominator.

**What this study adds?**

- Contemporary estimates of incidence for hospital admission for limb, chest, spinal and skull and facial fractures.
- The peak of upper limb fracture incidence is in 5–9 year-old children and for lower limb fractures it is in 10–15 year olds.
- Demonstration of stable or gradually reducing trends of admissions for most fractures except preschool skull fractures.

## Introduction

Fractures in children are a common presentation to the emergency department, with overall estimated annual incidence rates of 1500–3600 fractures per 100 000 children per year.^1–5^ Fractures in childhood cause pain and loss of opportunity.^6–8^ Most children contained within these series are managed in the community. However, some children require hospital admission and treatment. Admission to hospital is distressing, which is amplified if the child requires surgery,^9 10^ yet there is relatively little information regarding the numbers of children admitted for hospital treatment of fractures.

There have been several reports of decreased numbers of children presenting to hospitals with fractures during lockdown restrictions due to the coronavirus pandemic.^11 12^ These studies typically report absolute numbers of children and are not able to calculate rates of fractures due to uncertainty regarding the population size or denominator number. An evaluation of injury burden and trends in a population with a known denominator is important to allow for optimal service planning both in anticipation of removal of restrictions and for future years.

The National Health Service Hospital Episode Statistics (HES) is a national dataset detailing all completed consultant episodes in England. This includes emergency care where children are admitted to hospital. The dataset does not capture details of children who are treated exclusively in the community, or for whom a fracture is identified in the emergency department and then managed in a fracture clinic. This is therefore an ideal dataset to evaluate the volume of children admitted to hospitals in England as children with fractures are almost exclusively treated within the National Health Service (NHS).

The aim of this study is to evaluate the trends of children admitted to English hospitals since 2012. To do this, annual incidence of fractures will be calculated and analysed to identify current trends and by evaluating the trends in operative management of paediatric fractures during this period.

## Methods

Research ethics approval was not required for this study as it was a secondary analysis of publicly accessible data.

Open access records for finished consultant episodes were retrieved from NHS Digital from 2012 to 2020 on 20 February 2021 (https://digital.nhs.uk). Each consultant episode is coded by independent hospital coders with diagnoses from the International Classification of Diseases, 10th revision classification system and interventions were coded using the Office of Population Services and Censuses, 5th edition code book.^13 14^

The codes for acute fractures were classified into body regions (upper limb: clavicle, scapula, humerus, forearm, hand. Lower limb: pelvis, hip, femur, patella, lower leg and foot. Spine: cervical spine, thoracic spine, lumbar spine and sacral spine. Chest: sternum or ribs). Primary procedures were identified and classified according to type of reduction and fixation method (see online supplemental file 1). Patients without a primary diagnosis of fracture were excluded to minimise duplications.

10.1136/bmjpo-2021-001187.supp1 Supplementary data

The annual injury burden was calculated using the appropriate population estimate for different ages of children from the Office of National Statistics NOMIS service (https://www.nomisweb.co.uk/).

Incidence rates were calculated per 100 000 person years (PY) for all children and subdivided into four age groups (preschool 0–4, young children 5–9, older children 10–15 and adolescence 16–18). Annual trends were in incidence were visualised in Graphpad 7.04. Trends in injury incidence were fitted to a linear regression model with a significance level of <0.05.

### Patient involvement

Patient involvement was not deemed to be appropriate for the design or delivery of this study as an analysis of open-source data.

## Results

The population of children living in England has increased from 12 094 205 in 2012 to 12 642 441 in 2019. The trends in population change were not uniform across all age groups. There has been an increase in the population of children aged 5–9 and 10–15 years, a decrease in the population of children aged 16–18 and no significant change in the population of children aged 0–4 (table 1).

**Table 1:** Changes in demographics from the start and end of the study window

Age group	2012 population (% total)	2019 population (% total)	Annual change* (95% CI)	P value
Preschool (0–4)	3 393 356 (28.1)	3 299 637 (26.1)	−13 533 (−27 441 to 374)	0.055
Younger children (5–9)	3 083 582 (25.5)	3 538 206 (28.0)	66 762 (52 433 to 81 092)	<0.0001
Older children (10–15)	3 653 288 (30.2)	3 978 836 (31.5)	49 491 (20 402 to 78 580)	0.0059
Adolescents (16–18)	1 963 979 (16.2)	1 825 762 (14.4)	−20 960 (−26 060 to −15 861)	<0.0001

*Annual change calculated using a linear regression model.

During the study period, 368 120 admissions were recorded with a primary diagnosis of a fracture. These included 256 008 upper limb fractures, 85 737 lower limb fractures, 20 939 skull or facial fractures, 4542 spinal fractures and 894 chest fractures. The breakdown of these fractures into body area is shown in table 2. Across all age groups, upper limb fractures were the most frequent fracture type accounting for 26.2%–43.6% of fractures.

**Table 2:** Distribution of fractures admitted to hospitals in England between 2012 and 2019 according to age of child. Numbers shown are absolute numbers of consultant episodes and proportion of total fracture load

Body region	Preschool (age 0–4)	Younger children (age 5–9)	Older children (age 10–15)	Adolescents (age 16–18)
Upper limb	39 864 (32.7%)	93 648 (43.6%)	94 988 (34.8%)	27 508 (26.2%)
Forearm	18 507 (15.2%)	60 950 (28.4%)	62 797 (23.0%)	8576 (8.2%)
Humerus	14 820 (12.2%)	24 606 (11.5%)	8600 (3.2%)	1728 (1.6%)
Hand	3809 (3.1%)	7610 (3.5%)	20 940 (7.7%)	14 475 (13.8%)
Clavicle	2678 (2.2%)	399 (0.2%)	2403 (0.9%)	2544 (2.4%)
Scapula	9 (0.0%)	9 (0.0%)	100 (0.0%)	126 (0.1%)
Upper limb, unspecified	9 (0.0%)	58 (0.0%)	95 (0.0%)	22 (0.0%)
Shoulder, unspecified	32 (0.0%)	16 (0.0%)	53 (0.0%)	37 (0.0%)
Lower limb	17 292 (14.2%)	12 497 (5.8%)	37 622 (13.8%)	18 326 (17.4%)
Tibia/fibula	7670 (6.3%)	8088 (3.8%)	28 470 (0.4%)	12 501 (11.9%)
Femur	8382 (6.9%)	2804 (1.3%)	2991 (1.1%)	1856 (1.8%)
Foot (not calcaneum or talus)	613 (0.5%)	875 (0.4%)	2418 (0.9%)	1630 (1.6%)
Hip	522 (0.4%)	316 (0.1%)	1244 (0.5%)	346 (0.3%)
Pelvis	56 (0.0%)	143 (0.1%)	936 (0.0%)	848 (0.8%)
Patella	13 (0.0%)	166 (0.1%)	1126 (6.3%)	654 (0.6%)
Talus	10 (0.0%)	42 (0.0%)	239 (0.1%)	275 (0.3%)
Calcaneum	26 (0.0%)	63 (0.0%)	198 (0.1%)	216 (0.2%)
Spine	112 (0.1%)	249 (0.1%)	1451 (0.0%)	2730 (2.6%)
Thoracic spine	18 (0.0%)	106 (0.0%)	599 (0.2%)	911 (0.9%)
Lumbar spine	11 (0.0%)	69 (0.0%)	479 (0.2%)	1058 (1.0%)
Cervical spine	80 (0.1%)	56 (0.0%)	294 (0.1%)	555 (0.5%)
Sacral spine	3 (0.0%)	18 (0.0%)	79 (0.0%)	206 (0.2%)
Skull and facial bones	6970 (5.7%)	2025 (0.9%)	4303 (1.6%)	7641 (7.3%)
Chest	256 (0.2%)	64 (0.0%)	220 (0.1%)	354 (0.3%)

**Figure 1:**
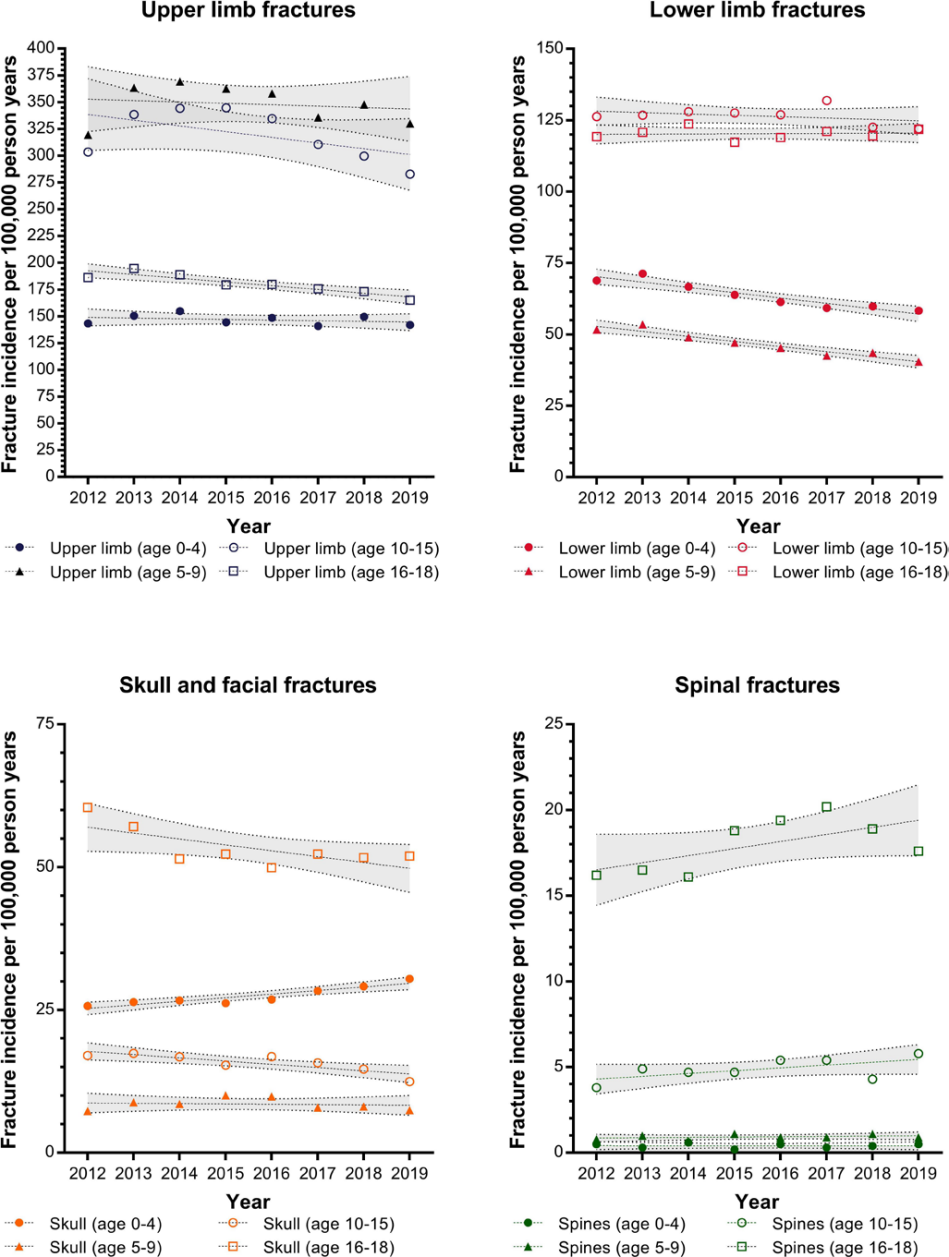
Trends of fracture incidence for upper limb, lower limb, skull and facial fractures and spinal fractures between 2012 and 2019. Data presented separated by the age groups of children with trends and 95% CIs calculated using a linear regression model.

Fracture incidence was calculated per 100 000 PY. The trends for upper limb, lower limb, skull and facial fractures and spinal fractures are shown in figure 1. Skull and facial fractures in children aged 0–4 were demonstrated to be increasing during the study window with an annual increase of 0.629 per 100 000 PY (95% CI 0.367 to 0.891, p=0.0011). The incidence was found to be decreasing for five groups of fractures. These are: (1) upper limb fractures in adolescents (annual decrease of 3.49 per 100 000 PY, 95% CI 1.944 to 5.036, p=0.0015); (2) lower limb fractures in preschool children (annual decrease 1.862 per 100 000 PY, 95% CI 1.222 to 2.502, p=0.0004); (3) lower limb fractures in younger children (annual decrease 1.773 per 100 000 PY, 95% CI 1.249 to 2.296, p=0.0002); (4) skull and facial fractures in adolescents (annual decrease 0.567 per 100 000 PY, 95% CI 0.208 to 0.926, p=0.0083) and (5) skull fractures in adolescents (annual decrease 1.03 per 100 000 PY, 95% CI 0.026 to 2.034, p=0.046). There was insufficient evidence to demonstrate an increase or decrease in incidence rates for other fracture types.

The incidence of different fractures is broken down by specific body region in table 3. In all age groups, the highest incidence of fractures was experienced in the upper limb with the forearm contributing the largest proportion of fractures in children aged 0–15. The peak incidence of forearm and humeral fractures was found in younger children while hand fractures were more common in older children. The lowest mean incidence of lower limb fractures was in younger children (46.7 per 100 000 PY). Femoral fractures were the most common lower limb fracture in preschool children, whereas tibial and ankle fractures were most common in all other age groups. The incidence of hip fractures is highest in older children and pelvic fractures highest in adolescents.

**Table 3:** Mean incidence of fractures per 100 000 person years (PY) admitted to hospitals in England between 2012 and 2019 according to age of child

Body region	Preschool (age 0–4)	Younger children (age 5–9)	Older children (age 10–15)	Adolescents (age 16–18)
Upper limb	146.9	348.3	319.9	180.4
Forearm	68.2	226.9	211.6	56.3
Humerus	54.6	91.3	28.9	11.3
Hand	14.0	28.3	70.5	94.9
Clavicle	9.9	1.5	8.1	16.7
Scapula	<0.1	<0.1	0/3	0.8
Upper limb, unspecified	<0.1	0.2	0.3	0.1
Shoulder, unspecified	0.1	0.1	0.2	0.2
Lower limb	63.7	46.7	126.5	120.3
Tibia/fibula	28.2	30.2	95.7	82.1
Femur	30.9	10.4	10.1	12.2
Foot (not calcaneum or talus)	2.3	3.3	8.1	10.7
Hip	1.9	1.2	4.2	2.3
Pelvis	0.2	0.5	3.1	5.6
Patella	<0.1	0.6	3.8	4.3
Talus	<0.1	0.2	0.8	1.8
Calcaneum	0.1	0.2	0.7	1.4
Spine	0.4	0.9	4.9	17.9
Thoracic spine	0.1	0.4	2.0	6.0
Lumbar spine	<0.1	0.3	1.6	7.0
Cervical spine	0.3	0.2	1.0	16.7
Sacral spine	<0.1	0.1	0.3	1.4
Skull and facial bones	25.7	7.5	14.5	50.1
Chest	0.9	0.2	0.7	2.3

Spine fractures were rare in preschool and younger children with a mean incidence of 0.4 per 100 000 PY for children aged 0–4 and 0.9 per 100 000 PY for children aged 5–9. There was a similar number of thoracic and lumber spine fractures. The highest incidence of cervical spine fractures was found in adolescents with a rate of 16.7 (95% CI 16.1 to 17.3) per 100 000 PY. This was higher than the combined rate of hip and femoral fractures.

The incidence of admissions for skull and facial bone fractures was lowest in younger children with a mean incidence of 7.5 per 100 000 PY for children aged 5–9. The highest incidence was for adolescents at 50.1 per 100 000 PY. Chest fractures (rib or sternum) were rare through all age groups with a maximum mean incidence of 2.3 per 100 000 PY for adolescents.

**Figure 2:**
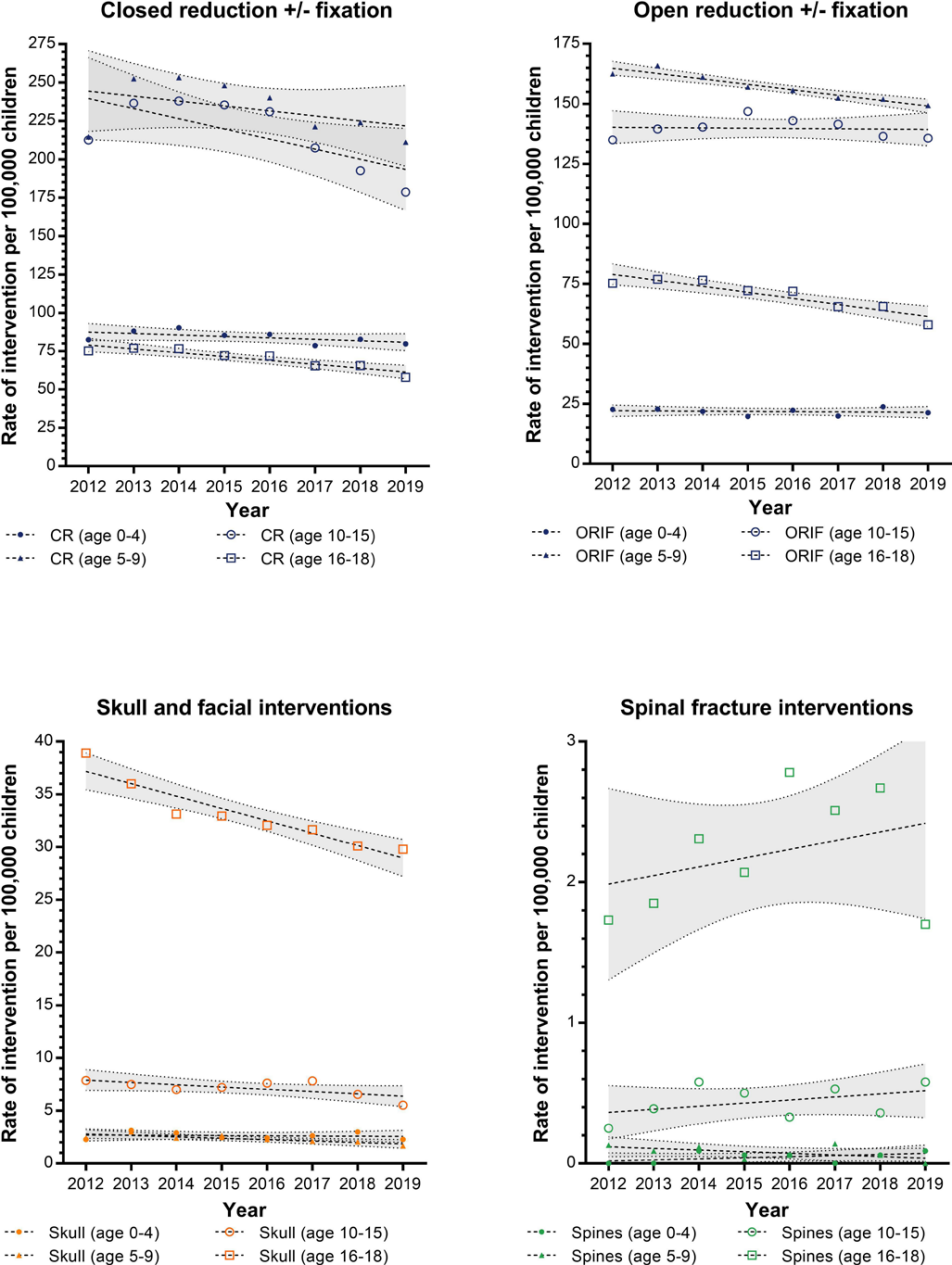
Trends of fracture interventions for limb open or closed reductions, skull and facial fractures and spinal fractures between 2012 and 2019. Data presented separated by the age groups of children with trends and 95% CIs calculated using a linear regression model. Abbreviations: CR closed reduction. ORIF open reduction internal fixation

The trends in the rates for surgical management of childhood fractures and head injuries are shown in figure 2. There was a peak of manipulative management of fractures seen between 2013 and 2016 in children aged 5–15 which mirrors the peak in admissions for upper limb fracture incidence identified in the same time period. Linear regression analysis identified a statistically significant decrease in the rates of open reduction and fixation in younger children (annual decrease of 2.3 per 100 000 PY, 95% CI 1.6 to 2.9, p=0.0002) and adolescents (annual decrease 2.5 per 100 000 PY, 95% CI 1.5 to 3.6, p=0.0010) and in closed reduction±fixation of older children (annual decrease 6.6 per 100 000 PY, 95% CI 0.2 to 13.0, p=0.0446) and adolescents (annual decrease 2.5, 95% CI 1.5 to 3.6, p=0.0010). A decrease in skull or facial fracture surgery was demonstrated for younger children with an annual decrease of 0.13 (95% CI 0.0 to 0.2) per 100 000 PY and for adolescents with an annual decrease of −1.2 (95% CI 0.8 to 1.6) per 100 000 PY. No change in operative rates was found for spinal surgery in any age group or skull and facial surgery in preschool children.

## Discussion

There is very little contemporary literature describing the trends of hospital admissions due to childhood fractures. This study has demonstrated trends in fracture patterns, distributions and treatment strategies that are valuable for exploration in future research and in planning healthcare prioritisation.

While the dataset used has several advantages, including the national coverage of all NHS funded admissions and procedures, the most significant limitation is a lack of granularity within this dataset beyond number of finished consultant episodes and numbers of procedures. The data do not extend to the community rates of fractures, or for fractures treated exclusively in the emergency department. However, they demonstrate a constant trend for most fractures, with a marginal decreasing trend in admissions for upper limb fractures in children aged 16–18, lower limb fractures in children aged 0–9 and skull fractures in children aged 10–18.

It is not clear what has been driving this decrease in limb and skull fracture admissions in these groups. It is possible that national safety campaigns are reducing the injury burden on hospitals. The Department of Transport reported that 2019 had the lowest number of child and young adult casualties and fatalities following road traffic accidents which may be associated with changing demographics of road users and the influence of safety campaigns including ‘Think!’.^15 16^ This may also be associated with an increase in sedentary lifestyles, though more work would be required to confirm this. Unfortunately, in the same time period, it has been identified that there has been a steady rise in penetrating trauma caused by knife crime, which with childhood admissions for knife wounds at the highest point in 2019 compared with 2012.^17^

The trend in upper limb fractures did not show a significant change. However, inspection of the graph shows that there was a peak in the number of admissions 2014–2015 which corresponds to a peak in the number of closed reductions. There has been an increasing awareness of non-surgical management of limb fractures and emergency department manipulation which avoid inpatient admission.^18–22^ Importantly, such fractures and their management would not be captured in this data.

The mean incidence of hospitalisation for childhood fractures was 374.8 per 100 000 children. This is lower than the 623.3 per 100 000 reported from Australia in the preceding decade (2002–2012).^23^ Part of this discrepancy may be that this previous study included admissions for nasal bone fractures, which contributed to 30% of the included facial fractures. The study from Australia also shows a decline in the number of admissions per 100 000 during the 10 years surveyed, which may have continued into the next decade.^23^ We have found a higher proportion of lower limb fractures admitted to hospitals in England than in the Australian cohort. This may be due to an increasing trend in the UK of management of many upper limb fractures in the community.

General practice databases have been used to develop estimates for fracture incidence in the UK. The Health Improvement Network research database from 495 of the 8228 general practitioners in the UK has been used to estimate the incidence of fractures in the under 5 population as 758 per 100 000 PY (95% CI 748–769). Moon *et al* used the Clinical Practice Research Datalink database from 1988 to 2012 (which covers 6.9% of the UK population) to estimate a national incidence of 1370 per 100 000 PY.^24 25^ Given that we have not found any major changes in the trends of fracture admissions since 2012 then it seems that approximately 30% of UK fractures result in hospital admission, however, the inpatient focus of the data for this study precludes a precise estimate.

A marginal increase in the incidence of admissions for preschool head and facial fractures was observed in the data. While mathematically significant, this represents an increase of only 19 admissions per year in a preschool population of 3.3 million. In this cohort, 83.5% of the fractures sustained by preschool children were to the base of skull or the skull vault with relatively few facial fractures. While this dataset provides insufficient granularity to evaluate the mechanism of injury, it is possible that this increase may represent an increase in diagnosis of fractures following non-accidental injury. Also, it could reflect changes in patterns of investigations, for example, skull CT, and management. Robust evidence for trends in non-accidental injury is difficult to obtain, but a rise in non-accidental injury has been documented in the USA with 95% of children involved age <5 years^26^.^27^ Alternatively, there may be an increased awareness of the impact of serious head injuries in this age group and thus a lower barrier to admissions driving this change.

This study has not included any hospital admissions from January 2020 onwards. The international coronavirus pandemic has been extensively documented to have caused significant disruption to emergency care, with many papers identifying fewer children attending emergency departments for treatment following fractures during the pandemic.^11 12 28–30^ This is likely to be due to lockdown policies limiting the availability of opportunities to participate in sport and adventurous play though in one series from London a reduction in non-surgical fractures was demonstrated without a corresponding decrease in surgical cases, suggesting that some more severe injuries were still occurring.^31^ What is unclear is how the rates of injuries will respond to the relaxation of lockdown rules over the next 12–24 months and if the suppressed number of admissions will continue or if there will be a rebound back to (or exceeding) the rates identified in this current study.

Due to the nature of the study design, there remain some limitations. The use of finished consultant episodes has been used previously to estimate the number of admissions attributed to injuries and musculoskeletal pathology.^32 33^ During the coding process, each patient episode is assigned up to 24 diagnostic codes. By analysing only primary diagnosis code, it is possible that the estimates in this study may have undercounted where patients with multiple injuries have presented. However, this strategy has allowed us to present the primary reason for admission, accepting this limitation. As for all database studies, there will be an error rate associated with the input of data into the HES registry. However, the HES database is externally audited and validated by NHS Digital, with 100% of finished consultant episodes being maintained with a primary diagnosis code in the included time period.^34^ In this period, many of these confounders would be expected to occur at a constant rate, and while there may be an impact on the absolute values the trend results should not be grossly impacted.

Despite these limitations, this study has shown a decreasing trend of admissions for many childhood fractures in most age groups, except preschool skull fractures and adolescent spinal fractures, which are increasing. These trends can be used to benchmark service provision for anticipated hospital volume following easing of lockdown restrictions if childhood behaviour returns to 2018–2019 activities. There is also a need to ensure adequate provision of trained trauma staff in emergency departments to provide skilled manipulation to maintain and continue downward limb fracture admission trends. Additional work is required to evaluate the causes of the trends observed and to develop safety strategies to safeguard infants and adolescents from these injuries.

## Supplementary Material

Author's manuscript

## Data Availability

Data are available upon reasonable request. Data used in calculation of incidence is available at request from the corresponding author.
