# Supplementary material for: Trends in hospital admissions for childhood fractures in England
Source: BMJ Paediatr Open. 2021 Nov 9;5(1):e001187. doi: 10.1136/bmjpo-2021-001187 (PMC8587381; doi:10.1136/bmjpo-2021-001187)
Supplement: Author's manuscript [file bmjpo-2021-001187.draft_revisions.pdf]

BMJ Paediatrics Open

BMJ Paediatrics Open is committed to open peer review. As part of this commitment we make the peer review history of every article we publish publicly available.

When an article is published we post the peer reviewers' comments and the authors' responses online. We also post the versions of the paper that were used during peer review. These are the versions that the peer review comments apply to.

The versions of the paper that follow are the versions that were submitted during the peer review process. They are not the versions of record or the final published versions. They should not be cited or distributed as the published version of this manuscript.

BMJ Paediatrics Open is an open access journal and the full, final, typeset and author-corrected version of record of the manuscript is available on our site with no access controls, subscription charges or pay-per-view fees (http://bmjpaedsopen.bmj.com).

If you have any questions on BMJ Paediatrics Open's open peer review process please email info.bmjpo@bmj.com

BMJ Paediatrics Open

Trends in childhood fractures in England between 2012-2019

Journal:	*BMJ Paediatrics Open*
Manuscript ID	bmjpo-2021-001187
Article Type:	Original research
Date Submitted by the Author:	13-Jun-2021
Complete List of Authors:	Marson, Ben; University of Nottingham School of Medicine, Orthopaedics and Trauma Group<br>Manning, Joseph ; Nottingham University Hospitals NHS Trust, Nottingham Children's Hospital; University of Nottingham, Children and Young people Health Research, School of Health Sciences<br>James, Marilyn; University of Nottingham School of Medicine, Clinical Trials Unit<br>Ikram, Adeel; University of Nottingham School of Medicine, Orthopaedics and Trauma Group<br>Bryson, David; Nottingham University Hospitals NHS Trust, Nottingham Children's Hospital<br>Ollivere, Benjamin; University of Nottingham School of Medicine, Orthopaedics and Trauma Group
Keywords:	Epidemiology

SCHOLARONE™
Manuscripts

I, the Submitting Author has the right to grant and does grant on behalf of all authors of the Work (as defined in the below author licence), an exclusive licence and/or a non-exclusive licence for contributions from authors who are: i) UK Crown employees; ii) where BMJ has agreed a CC-BY licence shall apply, and/or iii) in accordance with the terms applicable for US Federal Government officers or employees acting as part of their official duties; on a worldwide, perpetual, irrevocable, royalty-free basis to BMJ Publishing Group Ltd ("BMJ") its licensees and where the relevant Journal is co-owned by BMJ to the co-owners of the Journal, to publish the Work in this journal and any other BMJ products and to exploit all rights, as set out in our licence.

The Submitting Author accepts and understands that any supply made under these terms is made by BMJ to the Submitting Author unless you are acting as an employee on behalf of your employer or a postgraduate student of an affiliated institution which is paying any applicable article publishing charge ("APC") for Open Access articles. Where the Submitting Author wishes to make the Work available on an Open Access basis (and intends to pay the relevant APC), the terms of reuse of such Open Access shall be governed by a Creative Commons licence – details of these licences and which Creative Commons licence will apply to this Work are set out in our licence referred to above.

Other than as permitted in any relevant BMJ Author's Self Archiving Policies, I confirm this Work has not been accepted for publication elsewhere, is not being considered for publication elsewhere and does not duplicate material already published. I confirm all authors consent to publication of this Work and authorise the granting of this licence.

Confidential: For Review Only

Trends in childhood fractures in England between 2012-2019

Word count (2494 words)

Marson BA, NIHR Doctoral Fellow, Department of Trauma and Orthopaedics, University of Nottingham NG7 2UH. Ben.marson@nottingham.ac.uk

Manning JC, Associate Professor, School of Health Sciences, University of Nottingham, NG7 2UH

James M Professor of Health Economics Faculty of Medicine & Health Sciences, University of Nottingham, NG7 2UH

Ikram A, Doctoral Fellow, Department of Trauma and Orthopaedics, University of Nottingham NG7 2UH

Bryson DJ, Consultant in Paediatric Orthopaedics. Nottingham University Hospitals NHS trust, QMC, Nottingham, NG7 2UH

Ollivere BJ, Professor of Trauma Department of Trauma and Orthopaedics, University of Nottingham NG7 2UH

The Corresponding Author has the right to grant on behalf of all authors and does grant on behalf of all authors, an exclusive licence (or non exclusive for government employees) on a worldwide basis to the BMJ Publishing Group Ltd to permit this article (if accepted) to be published in BMJ editions and any other BMJPGL products and sublicences such use and exploit all subsidiary rights, as set out in our licence.

The manuscript guarantor (BJO) affirms that this manuscript is an honest, accurate, and transparent account of the study being reported; that no important aspects of the study have been omitted; and that any discrepancies from the study as planned (and, if relevant, registered) have been explained.

All authors have completed the ICMJE uniform disclosure at www.icmje.org/coi_disclosure.pdf and declare: financial support for the submitted work from NIHR; BAM has received a research grant from the NIHR to support this work. JM is funded through a NIHR grant unrelated to this work. Other authors have no support from any organisation for the submitted work; no financial relationships with any organisations that might have an interest in the submitted work in the previous three years; no other relationships or activities that could appear to have influenced the submitted work.

Confidential: For Review Only

Abstract

Purpose

Fractures to the axial and appendicular skeleton are common in children causing loss of opportunities and disability. There is relatively little data available to quantify the number of children who require hospital admission and treatment following these injuries, with most studies limited by a lack of robust denominator. The purpose of this study is to explore trends of frequency, types and age of children sustaining fractures requiring admission and intervention in NHS hospitals.

Design

The study uses data from the Hospital Episode Statistics and Office for National Statistics from 2012-2019 to calculate the annual incidence of upper limb, lower limb, spine, facial and skull fractures per 100,000 children.

Results

During 2012-2019, 368,120 children were admitted to NHS hospitals in England with a fracture. 256,018 (69.5%) were upper limb, 85,737 (23.3%) were lower limb fractures and 20,939 (5.7%) were skull or facial fractures. The annual incidence of upper limb fractures was highest in children aged 5-9 (348.3 per 100,000 children) and the highest incidence of lower limb fractures was in children aged 10-15(126.5 per 100,000 children). The incidence of skull and facial fractures in preschool (age 0-4) children has been increasing at a rate of 0.629 per 100,000 children per year.

Implications

The annual incidence of fractures in children has been shown to be consistent for several fracture types between 2012-2019. An increasing trend of preschool skull fractures is concerning, though the study data does not have sufficient granularity to demonstrate if this is due to accidental or non-accidental causes.

What is already known on the topic.

1. Fractures in children are common with more hospital admissions for upper limb than lower limb fractures.
2. Previous estimates of hospital admissions following childhood fractures are limited by a lack of robust denominator.

What this study adds

1. Contemporary estimates of incidence for limb, chest, spinal and skull and facial fractures.
2. The peak of upper limb fracture incidence is in 5–9 year-old children and for lower limb fractures it is in 10-15 year olds.
3. Demonstration of stable or gradually reducing trends for most fractures except preschool skull fractures.

Confidential: For Review Only

Introduction

Fractures in children are known to be a common presentation to the emergency department, with estimated annual incidence rates of 150-360 fractures per 10,000 children per year[1-5]. Fractures in childhood cause pain, disability, and loss of opportunity[6-8]. Admission to hospital is distressing, which is amplified if the child has to undergo a procedure following anaesthetic in an operating theatre[9 10].

Many of the children with fractures contained within these series may be successfully managed in the community. Others require inpatient admission and treatment for pain relief, fracture reduction or surgery. There is relatively little contemporary information regarding the inpatient burden of paediatric fractures, with previous literature restricted to single body areas, specific mechanisms of injury.

There have been several reports of decreased numbers of children presenting to hospitals with fractures during lockdown restrictions due to the coronavirus pandemic[11 12]. These studies typically report absolute numbers of children who are using services and are not able to calculate generalisable rates of fractures due to uncertainty regarding the population size or denominator number. An up-to-date evaluation of injury burden and trends in a population with a known denominator is important to allow for optimal service planning both in anticipation of schools and sports clubs fully re-opening and for future years.

The National Health Service Hospital Episode Statistics (HES) is a national data set detailing all completed consultant episodes in England. This can include emergency care where children are admitted by a speciality team from the emergency department, day case or planned procedures. The data set does not capture details of children who are treated exclusively in the community, or for whom a fracture is identified in the Emergency Department and then managed in a fracture clinic. This is therefore an ideal data set to evaluate the volume of children admitted to hospitals in England as children with fractures are almost exclusively treated within the NHS, and there are robust population estimates for this group from the Office of National Statistics.

The aim of this study is to evaluate the trends of children admitted to English hospitals since 2012. To do this annual incidence of fractures will be calculated and analysed to identify current trends and by evaluating the trends in operative management of paediatric fractures during this period.

Confidential: For Review Only

Methods

Research ethics approval was not required for this study as it was a secondary analysis of publicly accessible data.

Open access records for finished consultant episodes were retrieved from NHS Digital from 2012-2020 on the 20/02/2021 (https://digital.nhs.uk). Each consultant episode is coded by independent hospital coders with diagnoses from the International Classification of Diseases, 10th revision (ICD-10) classification system and procedures or interventions coded using the Office of Population Services and Censuses, 5th edition (OPCS-4) code book[13 14].

The codes for acute fractures were compiled and classified into the following body regions (Upper limb: clavicle, scapula, humerus, forearm, hand. Lower limb: pelvis, hip, femur, patella, lower leg and foot. Spine: cervical spine, thoracic spine, lumbar spine and sacral spine. Chest: sternum or ribs). Primary procedures were identified and classified according to type of reduction (open or closed) and fixation method (no fixation, internal fixation or external fixation). To minimise duplication, only patients with a primary diagnosis of fracture were included. Further details on the codes included for each body region and procedure classification are supplied in supplementary materials.

The annual injury burden was calculated using the appropriate population estimate for different ages of children. This was obtained for England from the Office of National Statistics Nomis service (https://www.nomisweb.co.uk/).

Incidence rates were calculated per 100,000 person years (PY) for all children and subdivided into four age groups (preschool 0-4, young children 5-9, older children 10-15, and adolescence 16-18). Annual trends were in incidence were visualised in Graphpad 7.04. Trends in injury incidence were fitted to a linear regression model with a significance level of <0.05.

Patient involvement

Patient involvement was not deemed to be appropriate for the design or delivery of this study as an analysis of open-source data.

Confidential: For Review Only

Results

The population of children living in England has increased from 12,094,205 in 2012 to 12,642,441 in 2019. The trends in population change were modelled in a linear regression which demonstrated the change in population has not been uniform across all age groups. There has been an increase in the population of children aged 5-9 and 10-15 years, a decrease in the population of children aged 16-18 and no significant change in the population of children aged 0-4 (Table 1).

During the study period, 368,120 admissions were recorded with a primary diagnosis of a fracture. These included 256,008 upper limb fractures, 85,737 lower limb fractures, 20,939 skull or facial fractures, 4,542 spinal fractures and 894 chest fractures. The breakdown of these fractures into body area is shown in Table 2. Across all age groups, upper limb fractures were the most frequent fracture type accounting for 26.2-43.6% of fractures.

Fracture incidence was calculated per 100,000 person years. The trends for upper limb, lower limb, skull and facial fractures and spinal fractures are shown in Figure 1. Skull and facial fractures in children aged 0-4 were demonstrated to be increasing during the study window with an annual increase of 0.629 per 100,000 PY (95% CI 0.367-0.891 p=0.0011). The incidence was found to be decreasing for five groups of fractures. These are: 1) upper limb fractures in children aged 16-18 (annual decrease of 3.49 per 100,000 PY, 95% CI 1.944-5.036 p=0.0015). 2) Lower limb fractures in children aged 0-4 (annual decrease 1.862 per 100,000 PY, 95% CI 1.222-2.502 p=0.0004). 3) lower limb fractures in children aged 5-9 (annual decrease 1.773 per 100,000 PY, 95% CI 1.249-2.296 p=0.0002). 4) skull and facial fractures in children aged 10-15 (annual decrease 0.567 per 100,000 PY, 95% CI 0.208-0.926 p=0.0083) and 5) skull fractures in children aged 16-18 (annual decrease 1.03 per 100,000 PY 95% CI 0.026-2.034 p=0.046). There was insufficient evidence to demonstrate an increase or decrease in incidence rates for other fracture types.

The incidence of different fractures is broken down by specific body region in Table 3. In all age groups, the highest incidence of fractures was experienced in the upper limb with the forearm contributing the largest proportion of fractures in children aged 0-15. The peak incidence of forearm and humeral fractures was found in children aged 5-9 while hand fractures were more common in older children. The lowest mean incidence of lower limb fractures was in children aged 5-9 (46.7 per 100,000PY) Femoral fractures were the most common lower limb fracture in children aged 0-4, whereas tibial and ankle fractures were most common in children aged 5-18. The incidence of hip fractures is highest in children aged 10-15 and pelvic fractures highest in children aged 16-18.

Spine fractures were rare in children aged 0-9 with a mean incidence of 0.4 (95% CI 0.3-0.5) and 0.9 (95% CI 0.8-1.0) per 100,000 PY. There was a similar number of thoracic and lumber spine fractures. The highest incidence of cervical spine fractures was found in children aged 16-18 with a rate of 16.7 (95% CI 16.1-17.3) per 100,000 PY. This was higher than the combined rate of hip and femoral fractures.

The incidence of skull and facial bone fractures was lowest in children aged 5-9 with a mean incidence of 25.7 (95% CI 24.4-27.0) per 100,000PY for children aged 0-4. The highest incidence was for children aged 16-18 at 50.1 (95% CI 47.2-53.0) per 100,000 PY. Chest fractures (rib or sternum) were rare through all age groups with a maximum mean incidence of 2.3 (95% CI 1.9-2.8) per 100,000PY for children aged 16-18.

The trends in the rates for surgical management of childhood fractures and head injuries is shown in Figure 2. There was a peak of manipulative management of fractures seen between 2013-2016 in children aged 5-15 which mirrors the peak in upper limb fracture incidence identified in the same

Confidential: For Review Only

time period. Linear regression analysis identified a statistically significant decrease in the rates of open reduction and fixation in children aged 5-9 (annual decrease of 2.3 per 100,000 PY 95% CI 1.6-2.9 p=0.0002) and 16-18 (annual decrease 2.5 per 100,000 PY, 95% CI 1.5-3.6 p=0.0010) and in closed reduction +/- fixation of children aged 10-15 (annual decrease 6.6 per 100,000PY, 95% CI 0.2-13.0 p=0.0446) and 16-18 (annual decrease 2.5, 95% CI 1.5-3.6 p=0.0010). A decrease in skull or facial fracture surgery was demonstrated for children aged 5-9 with an annual decrease of 0.13 (95% CI 0.0-0.2) per 100,000 PY and for children aged 16-18 with an annual decrease of -1.2 (95% CI 0.8-1.6) per 100,000 PY. No change in operative rates were found for spinal surgery in any age group or skull and facial surgery in preschool children.

Confidential: For Review Only

Table 1 Changes in demographics from the start and end of the study window. †Annual change calculated using a linear regression model

Age group	2012 population (% total)	2019 population (% total)	Annual change† (95% CI)	P value
0-4	3,393,356 (28.1%)	3,299,637 (26.1%)	-13533 (-27441 - 374)	0.055
5-9	3,083,582 (25.5%)	3,538,206 (28.0%)	66762 (52433 - 81092)	<0.0001
10-15	3,653,288 (30.2%)	3,978,836 (31.5%)	49491 (20402 - 78580)	0.0059
16-18	1,963,979 (16.2%)	1,825,762 (14.4%)	-20960 (-26060 - -15861)	<0.0001

Table 2 Distribution of fractures admitted to hospitals in England between 2012-2019 according to age of child. Numbers shown are absolute numbers of consultant episodes and proportion of total fracture load.

Body region	Age 0-4	Age 5-9	Age 10-15	Age 16-18
Upper limb	39,864 (32.7%)	93,648 (43.6%)	94,988 (34.8%)	27,508 (26.2%)
Forearm	*18,507 (15.2%)*	*60,950 (28.4%)*	*62,797 (23.0%)*	*8,576 (8.2%)*
Humerus	*14,820 (12.2%)*	*24,606 (11.5%)*	*8,600 (3.2%)*	*1,728 (1.6%)*
Hand	*3,809 (3.1%)*	*7,610 (3.5%)*	*20,940 (7.7%)*	*14,475 (13.8%)*
Clavicle	*2,678 (2.2%)*	*399 (0.2%)*	*2,403 (0.9%)*	*2,544 (2.4%)*
Scapula	*9 (0.0%)*	*9 (0.0%)*	*100 (0.0%)*	*126 (0.1%)*
Upper limb, unspecified	*9 (0.0%)*	*58 (0.0%)*	*95 (0.0%)*	*22(0.0%)*
Shoulder, unspecified	*32 (0.0%)*	*16 (0.0%)*	*53 (0.0%)*	*37(0.0%)*
Lower limb	17,292 (14.2%)	12,497 (5.8%)	37,622 (13.8%)	18,326 (17.4%)
Tibia/Fibula	*7,670 (6.3%)*	*8,088 (3.8%)*	*28,470 (0.4%)*	*12,501 (11.9%)*
Femur	*8,382 (6.9%)*	*2,804 (1.3%)*	*2,991 (1.1%)*	*1,856 (1.8%)*
Foot (not calcaneum or talus)	*613 (0.5%)*	*875 (0.4%)*	*2,418 (0.9%)*	*1,630 (1.6%)*
Hip	*522 (0.4%)*	*316 (0.1%)*	*1,244 (0.5%)*	*346 (0.3%)*
Pelvis	*56 (0.0%)*	*143 (0.1%)*	*936 (0.0%)*	*848 (0.8%)*
Patella	*13 (0.0%)*	*166 (0.1%)*	*1,126 (6.3%)*	*654 (0.6%)*
Talus	*10 (0.0%)*	*42 (0.0%)*	*239 (0.1%)*	*275 (0.3%)*
Calcaneum	*26 (0.0%)*	*63 (0.0%)*	*198 (0.1%)*	*216 (0.2%)*
Spine	112 (0.1%)	249 (0.1%)	1,451 (0.0%)	2,730 (2.6%)
Thoracic spine	*18 (0.0%)*	*106 (0.0%)*	*599 (0.2%)*	*911 (0.9%)*
Lumbar spine	*11 (0.0%)*	*69 (0.0%)*	*479 (0.2%)*	*1,058 (1.0%)*
Cervical spine	*80 (0.1%)*	*56 (0.0%)*	*294 (0.1%)*	*555 (0.5%)*
Sacral spine	*3 (0.0%)*	*18 (0.0%)*	*79 (0.0%)*	*206 (0.2%)*
Skull and facial bones	6,970 (5.7%)	2,025 (0.9%)	4,303 (1.6%)	7,641 (7.3%)
Chest	256 (0.2%)	64 (0.0%)	220 (0.1%)	354 (0.3%)

Figure 1 Trends of fracture incidence for upper limb, lower limb, skull and facial fractures and spinal fractures between 2012-2019. Data presented separated by the age groups of children with trends and 95% confidence intervals calculated using a linear regression model.

Confidential: For Review Only

Confidential: For Review Only

Table 3 Mean incidence of fractures per 100,000 person years (PY) admitted to hospitals in England between 2012-2019 according to age of child.

Body region	Age 0-4 (95% CI)	Age 5-9 (95% CI)	Age 10-15 (95% CI)	Age 16-18 (95% CI)
Upper limb	146.9 (142.9-150.9)	348.3 (333.2-363.4)	319.9 (300.2-339.6)	180.4 (173-188.2)
Forearm	*68.2 (64.8-71.6)*	*226.9 (213.5-240.4)*	*211.6 (195.9-227.3)*	*56.3 (53.4-59.1)*
Humerus	*54.6 (53.2-56.1)*	*91.3 (86.2-96.4)*	*28.9 (27.0-30.9)*	*11.3 (10.4-12.2)*
Hand	*14.0 (13.2-14.9)*	*28.3 (27.4-28.8)*	*70.5 (67.9-73.0)*	*94.9 (90.1-99.7)*
Clavicle	*9.9 (9.4-10.3)*	*1.5 (1.2-1.7)*	*8.1 (7.2-9.0)*	*16.7 (16.1-17.3)*
Scapula	*0.0 (0.0-0.1)*	*0.0 (0.0-0.1)*	*0.3 (0.3-0.4)*	*0.8 (0.6-1.1)*
Upper limb, unspecified	*0.0 (0.0-0.1)*	*0.2 (0.2-0.3)*	*0.3 (0.3-0.4)*	*0.1 (0.1-0.2)*
Shoulder, unspecified	*0.1 (0.1-0.2)*	*0.1 (0.0-0.1)*	*0.2 (0.1-0.2)*	*0.2 (0.1-0.4)*
Lower limb	63.7 (59.7-67.7)	46.7 (42.9-50.4)	126.5 (123.9-129.1)	120.3 (118.7-122.0)
Tibia/Fibula	*28.2 (25.1-31.4)*	*30.2 (27.0-33.3)*	*95.7 (93.5-98.0)*	*82.1 (80.4-84.8)*
Femur	*30.9 (30.2-31.6)*	*10.4 (9.8-11.1)*	*10.1 (9.7-10.4)*	*12.2 (11.2-13.2)*
Foot (not calcaneum or talus)	*2.3 (2.0-2.5)*	*3.3 (2.9-3.7)*	*8.1 (7.6-8.6)*	*10.7 (9.6-11.8)*
Hip	*1.9 (1.7-2.2)*	*1.2 (1.0-1.4)*	*4.2 (3.9-4.5)*	*2.3 (2.1-2.5)*
Pelvis	*0.2 (0.1-0.3)*	*0.5 (0.4-0.7)*	*3.1 (2.9-3.4)*	*5.6 (5.0-6.2)*
Patella	*0.0 (0.0-0.1)*	*0.6 (0.5-0.8)*	*3.8 (3.4-4.1)*	*4.3 (3.8-4.8)*
Talus	*0.0 (0.0-0.1)*	*0.2 (0.1-2.1)*	*0.8 (0.7-0.9)*	*1.8 (1.5-2.1)*
Calcaneum	*0.1 (0.0-0.2)*	*0.2 (0.2-0.3)*	*0.7 (0.5-0.8)*	*1.4 (1.2-1.7)*
Spine	0.4 (0.3-0.5)	0.9 (0.8-1.0)	4.9 (4.3-5.4)	17.9 (16.6-19.3)
Thoracic spine	*0.1 (0.0-0.1)*	*0.4 (0.3-0.5)*	*2.0 (1.8-2.3)*	*6.0 (5.3-6.7)*
Lumbar spine	*0.0 (0.0-0.1)*	*0.3 (0.2-0.3)*	*1.6 (1.3-1.9)*	*7.0 (6.3-7.7)*
Cervical spine	*0.3 (0.2-0.4)*	*0.2 (0.2-0.3)*	*1.0 (0.8-1.2)*	*16.7 (16.1-17.3)*
Sacral spine	*0.0 (0.0-0.3)*	*0.1 (0.0-0.1)*	*0.3 (0.2-0.3)*	*1.4 (1.1-1.6)*
Skull and facial bones	25.7 (24.4-27.0)	7.5 (6.7-8.3)	14.5 (13.15.8)	50.1 (47.2-53.0)
Chest	0.9 (0.7-1.2)	0.2 (0.2-0.3)	0.7 (0.6-0.9)	2.3 (1.9-2.8)

Figure 2 Trends of fracture interventions for limb open or closed reductions, skull and facial fractures and spinal fractures between 2012-2019. Data presented separated by the age groups of children with trends and 95% confidence intervals calculated using a linear regression model.

Confidential: For Review Only

Discussion

There is very little contemporary literature describing the trends of hospital admissions due to axial and appendicular fractures. This study has demonstrated some important trends in fracture patterns, distributions and treatment strategies that are valuable for exploration in future research and in planning healthcare prioritisation.

While the dataset used has several advantages, including the national coverage of all NHS funded admissions and procedures, the most significant limitation is a lack of granularity within this dataset beyond number of finished consultant episodes and numbers of procedures. Despite this, the data demonstrates a decreasing trend in upper limb fractures in children aged 16-18, lower limb fractures in children aged 0-9, and skull fractures in children aged 10-18.

It is not clear what has been driving this decrease in limb and skull fractures in this age range. It is likely that multiple safety campaigns are reducing the injury burden on hospitals. The Department of Transport reported that 2019 had the lowest number of child and young adult casualties and fatalities following road traffic accidents which may be associated with changing demographics of road users and the influence of safety campaigns such as Think![15 16]. This may also be associated with an increase in sedentary lifestyles, though more work would be required to confirm this. Unfortunately, in the same time period, it has been identified that there has been a steady rise in penetrating trauma caused by knife crime, which with childhood admissions for knife wounds at the highest point in 2019 compared to 2012[17].

The trend in upper limb fractures did not show a significant change with linear regression. However, inspection of the graph shows that there was a peak in the number of admissions 2014-2015 which corresponds to a peak in the number of closed reductions. There has been an increasing awareness of non-surgical management of some upper limb fractures and manipulation under analgesia or sedation which avoid inpatient admission and theatre procedures[18-20].

The overall mean incidence of hospitalisation for childhood fractures was 374.8 per 100,000 children. This is lower than the 623.3 per 100,000 reported from Australia in the preceding decade (2002-2012). Part of this discrepancy may be that this previous study included admissions for nasal bone fractures, which contributed to 30% of the included facial fractures. The study from Australia also shows a decline in the number of admissions per 100,000 during the 10 years surveyed, which may have continued into the next decade[21]. We have found a higher proportion of lower limb fractures admitted to hospitals in England than in the Australian cohort. This may be due to an increasing trend in the U.K. of management of many upper limb fractures in the community.

General Practice databases have been used to develop estimates for fracture incidence in the United Kingdom. Orton et al used the Health Improvement Network (THIN) research database from 495 of the 8,228 general practitioners in the United Kingdom to estimate the incidence of fractures in the under 5 population of 758 per 100,000 PY (95% confidence interval 748–769) and Moon et al used the Clinical Practice Research Datalink (CPRD) database from 1988-2012 (which covers 6.9% of the UK population) to estimate a national incidence of 1,370 per 100,000 person years[22 23]. These studies highlight the significant additional burden of childhood fractures treated exclusively in the Emergency Department and fracture clinics that were not included in this present study.

Of potential concern is the rising number of admissions for preschool skull and facial fractures. In this cohort, 83.5% of the fractures sustained by preschool children were to the base of skull or the skull vault with relatively few facial fractures. While this data set provides insufficient granularity to evaluate the mechanism of injury, it is possible that this increase may represent an increase in

Confidential: For Review Only

diagnosis of fractures following non-accidental injury. Robust evidence for trends in non-accidental injury is difficult to obtain, but a rising incidence of non-accidental injury has been documented in the United States with 95% of children involved age <5[24 25].

This study has not included any hospital admissions from January 2020 onwards. The international coronavirus pandemic has been extensively documented to have caused significant disruption to Emergency care, with many papers identifying fewer children attending Emergency Departments for treatment following fractures during the pandemic[11 12 26-28]. This is likely to be due to lockdown policies limiting the availability of opportunities to participate in sport and adventurous play though in one series from London a reduction in non-surgical fractures was demonstrated without a corresponding decrease in surgical cases, suggesting that some more severe injuries were still occurring[29]. What is unclear is how the rates of injuries will respond to the relaxation of lockdown rules over the next 12-24 months and if the suppressed number of admissions will continue or if there will be a rebound back to (or exceeding) the rates identified in this current study.

Due to the nature of the study design, there remains some limitations. The use of finished consultant episodes has been used previously to estimate the number of admissions attributed to injuries or other musculoskeletal pathology.[30 31] During the coding process, each patient episode is assigned up to 24 diagnostic codes. By analysing only primary diagnosis code it is possible that the estimates in this study may have undercounted where patients with multiple injuries have presented. However, this strategy has allowed us to present the primary reason for admission, accepting this limitation. As for all database studies, there will be an error rate associated with the input of data into the HES registry. However, the HES database is externally audited and validated by NHS Digital, with one hundred percent of finished consultant episodes being maintained with a primary diagnosis code in the included time period[32].

Despite these limitations, this study has shown a decreasing trend for many limb and skull fractures in most age groups, except preschool skull fractures and adolescent spinal fractures, which are increasing. These trends can be used to benchmark service provision for anticipated hospital volume following easing of lockdown restrictions if childhood behaviour returns to 2018-2019 activities. There is also a need to ensure adequate provision of trained trauma staff in Emergency Departments to provide skilled manipulation to maintain and continue downward limb fracture admission trends. Additional work is required to evaluate the causes of the trends observed and to develop safety strategies to safeguard infants and adolescents from these injuries.

Confidential: For Review Only

1. Landin LA. Fracture patterns in children. Analysis of 8,682 fractures with special reference to incidence, etiology and secular changes in a Swedish urban population 1950-1979. *Acta orthopaedica Scandinavica Supplementum* 1983;202:1-109. [published Online First: 1983/01/01]
2. Tiderius CJ, Landin L, Düppe H. Decreasing incidence of fractures in children: an epidemiological analysis of 1,673 fractures in Malmö, Sweden, 1993-1994. *Acta Orthop Scand* 1999;70(6):622-6. doi: 10.3109/17453679908997853 [published Online First: 2000/02/09]
3. Kang MS, Kim HS. Characteristics and trends of traumatic injuries in children visiting emergency departments in South Korea: A retrospective serial cross-sectional study using both nationwide-sample and single-institutional data. *PLoS One* 2019;14(8):e0220798. doi: 10.1371/journal.pone.0220798 [published Online First: 2019/08/10]
4. Bergman E, Lempesis V, Nilsson J-Å, et al. Time trends in pediatric fractures in a Swedish city from 1950 to 2016. *Acta Orthopaedica* 2020;91(5):598-604. doi: 10.1080/17453674.2020.1783484
5. Orces CH, Orces J. Trends in the U.S. Childhood Emergency Department Visits for Fall-Related Fractures, 2001-2015. *Cureus* 2020;12(11):e11629. doi: 10.7759/cureus.11629 [published Online First: 2020/12/31]
6. Ding R, McCarthy ML, Houseknecht E, et al. The health-related quality of life of children with an extremity fracture: a one-year follow-up study. *J Pediatr Orthop* 2006;26(2):157-63. doi: 10.1097/01.bpo.0000218521.98244.7e [published Online First: 2006/03/25]
7. Kendrick D, Vinogradova Y, Coupland C, et al. Recovery from injury: the UK burden of injury multicentre longitudinal study. *Injury prevention* 2013;19(6):370-81.
8. Schneeberg A, Ishikawa T, Kruse S, et al. A longitudinal study on quality of life after injury in children. *Health and quality of life outcomes* 2016;14(1):120.
9. FANZCA M. Distress at induction of anaesthesia in children. A survey of incidence, associated factors and recovery characteristics. *Pediatric Anesthesia* 1998;8(5):383-92.
10. Bentley J. Distress in children attending A&E. *Emergency nurse* 2004;12(4)
11. Murphy T, Akehurst H, Mutimer J. Impact of the 2020 COVID-19 pandemic on the workload of the orthopaedic service in a busy UK district general hospital. *Injury* 2020;51(10):2142-47.
12. Sugand K, Park C, Morgan C, et al. Impact of the COVID-19 pandemic on paediatric orthopaedic trauma workload in central London: a multi-centre longitudinal observational study over the "golden weeks". *Acta Orthop* 2020;91(6):633-38. doi: 10.1080/17453674.2020.1807092 [published Online First: 2020/08/25]
13. Digital N. OPCS Classification of Interventions and Procedures 2021 [Available from: https://datadictionary.nhs.uk/supporting_information/opcs_classification_of_interventions_and_procedures.html accessed 30/4/2021.
14. Organization WH. ICD-10 : international statistical classification of diseases and related health problems : tenth revision. 2nd ed. Geneva: World Health Organization. , 2004.
15. Murphy A. Reported road casualties in Great Britain: 2019 annual report. London: Department for Transport, 2020.
16. Transport Df. Think! 2021 [Available from: https://www.think.gov.uk/ accessed 30/4/2021.
17. Allen G, Kirk-Wade E. Knife crime in England and Wales. Briefing Paper. London: House of Commons Library, 2020.
18. Kurien T, Price K, Pearson RG, et al. Manipulation and reduction of paediatric fractures of the distal radius and forearm using intranasal diamorphine and 50% oxygen and nitrous oxide in the emergency department: a 2.5-year study. *The bone & joint journal* 2016;98(1):131-36.
19. Marson BA, Ng JW, Craxford S, et al. Treatment of completely displaced distal radial fractures with a straight plaster or manipulation under anaesthesia. *The Bone & Joint Journal* 2021:1-6.
20. Patel D, Talbot C, Luo W, et al. The use of esketamine sedation in the emergency department for manipulation of paediatric forearm fractures: A 5 year study. *Injury* 2021

Confidential: For Review Only

21. Faris M, Lystad RP, Harris I, et al. Fracture-related hospitalisations and readmissions of Australian children ≤16 years: A 10-year population-based cohort study. *Injury* 2020;51(10):2172-78. doi: 10.1016/j.injury.2020.07.032 [published Online First: 2020/07/28]
22. Moon RJ, Harvey NC, Curtis EM, et al. Ethnic and geographic variations in the epidemiology of childhood fractures in the United Kingdom. *Bone* 2016;85:9-14. doi: 10.1016/j.bone.2016.01.015 [published Online First: 2016/01/24]
23. Orton E, Kendrick D, West J, et al. Persistence of health inequalities in childhood injury in the UK: a population-based cohort study of children under 5. *PLoS One* 2014;9(10):e111631. doi: 10.1371/journal.pone.0111631 [published Online First: 2014/10/28]
24. Rosenfeld EH, Johnson B, Wesson DE, et al. Understanding non-accidental trauma in the United States: A national trauma databank study. *Journal of Pediatric Surgery* 2020;55(4):693-97. doi: https://doi.org/10.1016/j.jpedsurg.2019.03.024
25. Thompson LW, Bass K, Agyei J, et al. Rising Incidence of Non-Accidental Trauma in Infants: A Call to Revisit Prevention Strategies. *Pediatrics* 2018;142:749-49. doi: 10.1542/peds.142.1_MeetingAbstract.749
26. Bram JT, Johnson MA, Magee LC, et al. Where have all the fractures gone? The epidemiology of pediatric fractures during the COVID-19 pandemic. *Journal of Pediatric Orthopaedics* 2020;40(8):373-79.
27. Nabian MH, Vosoughi F, Najafi F, et al. Epidemiological pattern of pediatric trauma in COVID-19 outbreak: Data from a tertiary trauma center in Iran. *Injury* 2020;51(12):2811-15. doi: 10.1016/j.injury.2020.09.015 [published Online First: 2020/09/23]
28. Pines JM, Zocchi MS, Black BS, et al. Characterizing pediatric emergency department visits during the COVID-19 pandemic. *The American journal of emergency medicine* 2021;41:201-04.
29. Ibrahim Y, Huq S, Shanmuganathan K, et al. Trampolines injuries are bouncing back: The effect of the COVID-19 UK lockdown on the paediatric trauma burden. *Bone & Joint Open* 2021;2(2):86.
30. Judge A, Murphy R, Maxwell R, et al. Temporal trends and geographical variation in the use of subacromial decompression and rotator cuff repair of the shoulder in England. *The bone & joint journal* 2014;96(1):70-74.
31. Manley OW, Wormald JC, Furniss D. The changing shape of hand trauma: an analysis of Hospital Episode Statistics in England. *Journal of hand surgery (European volume)* 2019;44(5):532-36.
32. Digital N. HES Data Quality Statement 2020 [Available from: https://digital.nhs.uk/data-and-information/publications/statistical/hospital-admitted-patient-care-activity/2019-20/data-quality-statement accessed 30/4/2021.

Confidential: For Review Only

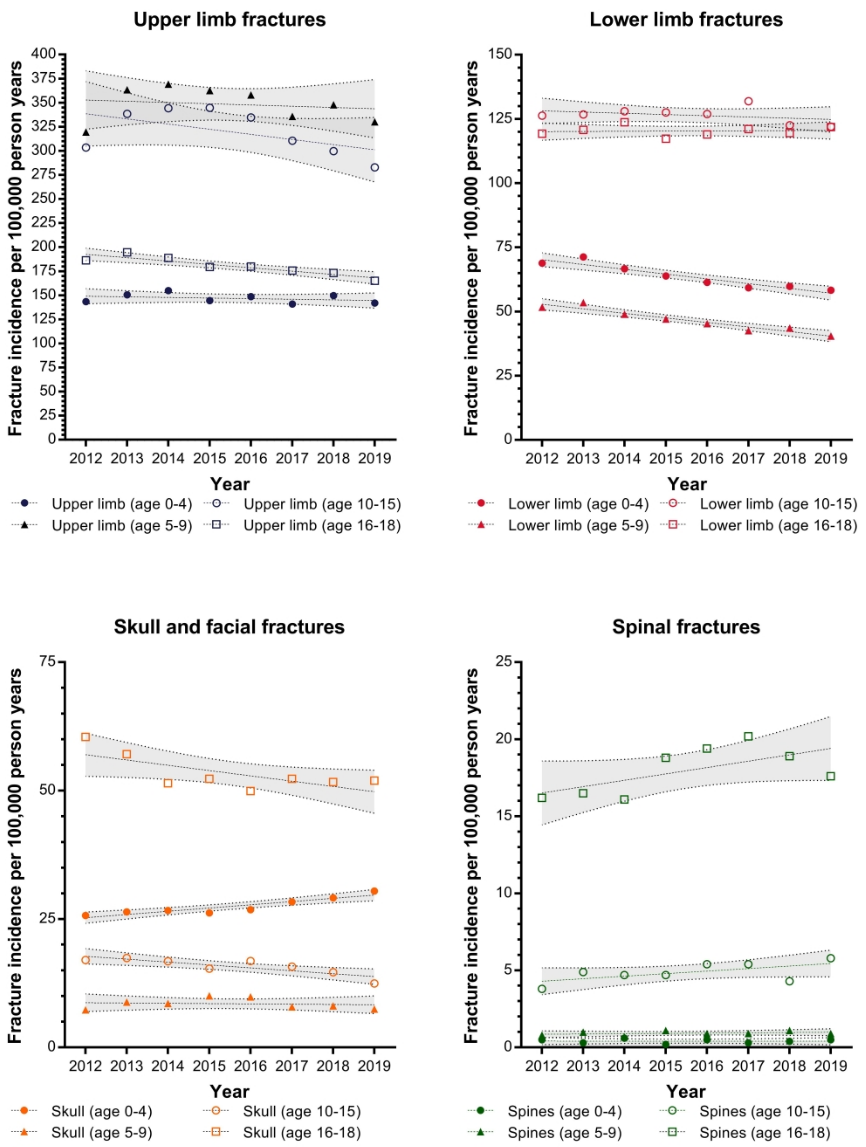

200x253mm (600 x 600 DPI)

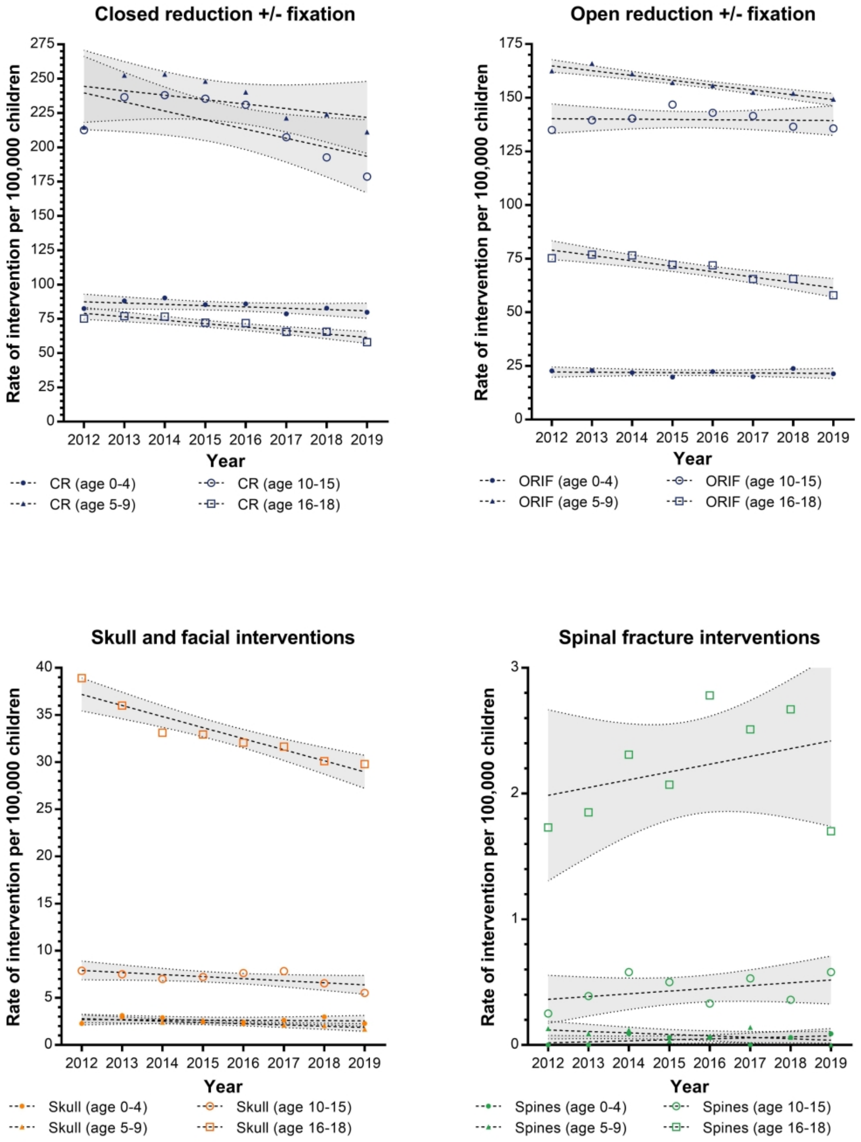

195x247mm (600 x 600 DPI)

List of included codes

code	Name	Mapped to body area
S12.0	Fracture of first cervical vertebra	C-spine
S12.1	Fracture of second cervical vertebra	C-spine
S12.2	Fracture of other specified cervical vertebra	C-spine
S12.8	Fracture of other parts of neck	C-spine
S12.9	Fracture of neck, part unspecified	C-spine
S62.0	Fracture of navicular [scaphoid] bone of han	carpus
S62.1	Fracture of other carpal bone(s)	carpus
P13.4	Fracture of clavicle due to birth injury	clavicle
S42.0	Fracture of clavicle	clavicle
S72.3	Fracture of shaft of femur	femur
S72.4	Fracture of lower end of femur	femur
S72.8	Fractures of other parts of femur	femur
S72.9	Fracture of femur, part unspecified	femur
S92.9	Fracture of foot, unspecified	foot
S52.0	Fracture of upper end of ulna	Forearm
S52.1	Fracture of upper end of radius	Forearm
S52.2	Fracture of shaft of ulna	Forearm
S52.3	Fracture of shaft of radius	Forearm
S52.4	Fracture of shafts of both ulna and radius	Forearm
S52.5	Fracture of lower end of radius	Forearm
S52.6	Fracture of lower end of both ulna and radiu	Forearm
S52.8	Fracture of other parts of forearm	Forearm
S52.9	Fracture of forearm, part unspecified	Forearm
S62.5	Fracture of thumb	hand
S62.6	Fracture of other finger	hand
S62.8	Fracture of other and unspecified parts of wr	hand
S92.0	Fracture of calcaneus	hindfoot
S92.1	Fracture of talus	hindfoot
S72.0	Fracture of neck of femur	hip
S72.1	Pertrochanteric fracture	hip
S72.2	Subtrochanteric fracture	hip
S42.2	Fracture of upper end of humerus	humerus
S42.3	Fracture of shaft of humerus	humerus
S42.4	Fracture of lower end of humerus	humerus
S32.0	Fracture of lumbar vertebra	L-spine
S82.1	Fracture of upper end of tibia	lower leg
S82.2	Fracture of shaft of tibia	lower leg
S82.3	Fracture of lower end of tibia	lower leg
S82.4	Fracture of fibula alone	lower leg
S82.5	Fracture of medial malleolus	lower leg
S82.6	Fracture of lateral malleolus	lower leg
S82.8	Fractures of other parts of lower leg	lower leg
S82.9	Fracture of lower leg, part unspecified	lower leg
S62.2	Fracture of first metacarpal bone	metacarp
S62.3	Fracture of other metacarpal bone	metacarp
S82.0	Fracture of patella	patella
S32.1	Fracture of sacrum	pelvis
S32.2	Fracture of coccyx	pelvis
S32.3	Fracture of ilium	pelvis
S32.4	Fracture of acetabulum	pelvis
S32.5	Fracture of pubis	pelvis
S32.8	Fracture of other and unspecified parts of lu	pelvis
S42.1	Fracture of scapula	scapula
P13.0	Fracture of skull due to birth injury	skull
S02.0	Fracture of vault of skull	skull
S02.1	Fracture of base of skull	skull
S02.3	Fracture of orbital floor	skull
S02.4	Fracture of malar and maxillary bones	skull
S02.6	Fracture of mandible	skull
S02.8	Fractures of other skull and facial bones	skull
S02.9	Fracture of skull and facial bones, part unsp	skull

Confidential: For Review Only

T08.X	Fracture of spine, level unspecified	spine
S22.0	Fracture of thoracic vertebra	t0spine
S92.2	Fracture of other tarsal bone(s)	tarsal
S92.3	Fracture of metatarsal bone	tarsal
S22.2	Fracture of sternum	thorax
S22.3	Fracture of rib	thorax
S22.8	Fracture of other parts of bony thorax	thorax
S22.9	Fracture of bony thorax, part unspecified	thorax
S92.4	Fracture of great toe	toe
S92.5	Fracture of other toe	toe

Confidential: For Review Only

BMJ Paediatrics Open

Trends in hospital admissions for childhood fractures in England

Journal:	*BMJ Paediatrics Open*
Manuscript ID	bmjpo-2021-001187.R1
Article Type:	Original research
Date Submitted by the Author:	23-Sep-2021
Complete List of Authors:	Marson, Ben; University of Nottingham School of Medicine, Orthopaedics and Trauma Group<br>Manning, Joseph ; Nottingham University Hospitals NHS Trust, Nottingham Children's Hospital; University of Nottingham, Children and Young people Health Research, School of Health Sciences<br>James, Marilyn; University of Nottingham School of Medicine, Clinical Trials Unit<br>Ikram, Adeel; University of Nottingham School of Medicine, Orthopaedics and Trauma Group<br>Bryson, David; Nottingham University Hospitals NHS Trust, Nottingham Children's Hospital<br>Ollivere, Benjamin; University of Nottingham School of Medicine, Orthopaedics and Trauma Group
Keywords:	Epidemiology

SCHOLARONE™
Manuscripts

I, the Submitting Author has the right to grant and does grant on behalf of all authors of the Work (as defined in the below author licence), an exclusive licence and/or a non-exclusive licence for contributions from authors who are: i) UK Crown employees; ii) where BMJ has agreed a CC-BY licence shall apply, and/or iii) in accordance with the terms applicable for US Federal Government officers or employees acting as part of their official duties; on a worldwide, perpetual, irrevocable, royalty-free basis to BMJ Publishing Group Ltd ("BMJ") its licensees and where the relevant Journal is co-owned by BMJ to the co-owners of the Journal, to publish the Work in this journal and any other BMJ products and to exploit all rights, as set out in our licence.

The Submitting Author accepts and understands that any supply made under these terms is made by BMJ to the Submitting Author unless you are acting as an employee on behalf of your employer or a postgraduate student of an affiliated institution which is paying any applicable article publishing charge ("APC") for Open Access articles. Where the Submitting Author wishes to make the Work available on an Open Access basis (and intends to pay the relevant APC), the terms of reuse of such Open Access shall be governed by a Creative Commons licence – details of these licences and which Creative Commons licence will apply to this Work are set out in our licence referred to above.

Other than as permitted in any relevant BMJ Author's Self Archiving Policies, I confirm this Work has not been accepted for publication elsewhere, is not being considered for publication elsewhere and does not duplicate material already published. I confirm all authors consent to publication of this Work and authorise the granting of this licence.

Confidential: For Review Only

Trends in hospital admissions for childhood fractures in England

Word count (2487 words)

Marson BA, NIHR Doctoral Fellow, Department of Trauma and Orthopaedics, University of Nottingham NG7 2UH. Ben.marson@nottingham.ac.uk

Manning JC, Associate Professor, School of Health Sciences, University of Nottingham, NG7 2UH

James M Professor of Health Economics Faculty of Medicine & Health Sciences, University of Nottingham, NG7 2UH

Ikram A, Doctoral Fellow, Department of Trauma and Orthopaedics, University of Nottingham NG7 2UHco.uk)

Bryson DJ, Consultant in Paediatric Orthopaedics. Nottingham University Hospitals NHS trust, QMC, Nottingham, NG7 2UH

Ollivere BJ, Professor of Trauma Department of Trauma and Orthopaedics, University of Nottingham NG7 2UH

The Corresponding Author has the right to grant on behalf of all authors and does grant on behalf of all authors, an exclusive licence (or non exclusive for government employees) on a worldwide basis to the BMJ Publishing Group Ltd to permit this article (if accepted) to be published in BMJ editions and any other BMJPGL products and sublicences such use and exploit all subsidiary rights, as set out in our licence.

The manuscript guarantor (BJO) affirms that this manuscript is an honest, accurate, and transparent account of the study being reported; that no important aspects of the study have been omitted; and that any discrepancies from the study as planned (and, if relevant, registered) have been explained.

All authors have completed the ICMJE uniform disclosure at www.icmje.org/coi_disclosure.pdf and declare: financial support for the submitted work from NIHR; BAM has received a research grant from the NIHR to support this work. JM is funded through a NIHR grant unrelated to this work. Other authors have no support from any organisation for the submitted work; no financial relationships with any organisations that might have an interest in the submitted work in the previous three years; no other relationships or activities that could appear to have influenced the submitted work.

BAM: Data acquisition, analysis, writing of first draft

JCM: Study conceptualisation, analysis, editing of final draft, supervision of trainee

MJ: Study conceptualisation, analysis, editing of final draft, supervision of trainee

AI: Data acquisition, analysis, editing of final draft

DJB: Study conceptualisation, analysis, contextualisation of study, editing of final draft, supervision of trainees

BJO: Study conceptualisation, analysis, editing of final draft, supervision of trainees. BJO is the guarantor of the study

Confidential: For Review Only

Abstract

Purpose

Fractures to the axial and appendicular skeleton are common in children causing loss of opportunities and disability. There is relatively little data available to quantify the number of children who have their fractures diagnosed in the Emergency Department and are then admitted to hospital for ongoing management. The purpose of this study is to explore trends of frequency, types and age of children sustaining fractures requiring admission and intervention in NHS hospitals.

Design

The study uses data from the Hospital Episode Statistics and Office for National Statistics from 2012-2019 to calculate the annual incidence of hospital admission for limb, spine, facial and skull fractures per 100,000 children.

Results

During 2012-2019, 368,120 children were admitted to English NHS hospitals with a fracture. 256,018 (69.5%) were upper limb, 85,737 (23.3%) were lower limb fractures and 20,939 (5.7%) were skull or facial fractures. The annual incidence of upper limb fractures was highest in children aged 5-9 (348.3 per 100,000 children) and the highest incidence of lower limb fractures was in children aged 10-15(126.5 per 100,000 children). The incidence of skull and facial fractures in preschool (age 0-4) children has been increasing at a rate of 0.629 per 100,000 children per year.

Implications

The annual incidence of hospital admission for fractures in children has been shown to be consistent for several fracture types between 2012-2019. An increasing trend of preschool skull fractures was observed, though the study data does not have sufficient granularity to demonstrate if this is due to accidental or non-accidental causes.

What is already known on the topic.

1. Fractures in children are common with more hospital admissions for upper limb than lower limb fractures.
2. Previous estimates of hospital admissions following childhood fractures are limited by a lack of robust denominator.

What this study adds

1. Contemporary estimates of incidence for hospital admission for limb, chest, spinal and skull and facial fractures.
2. The peak of upper limb fracture incidence is in 5–9 year-old children and for lower limb fractures it is in 10-15 year olds.
3. Demonstration of stable or gradually reducing trends for most fractures except preschool skull fractures.

Confidential: For Review Only

Introduction

Fractures in children are a common presentation to the Emergency Department, with overall estimated annual incidence rates of 1,500-3,600 fractures per 100,000 children per year[1-5]. Fractures in childhood cause pain and loss of opportunity[6-8]. Most children contained within these series are managed in the community. However, some children require hospital admission and treatment. Admission to hospital is distressing, which is amplified if the child requires surgery[9 10], yet there is relatively little information regarding the numbers of children admitted for hospital treatment of fractures.

There have been several reports of decreased numbers of children presenting to hospitals with fractures during lockdown restrictions due to the coronavirus pandemic[11 12]. These studies typically report absolute numbers of children and are not able to calculate rates of fractures due to uncertainty regarding the population size or denominator number. An evaluation of injury burden and trends in a population with a known denominator is important to allow for optimal service planning both in anticipation of removal of restrictions and for future years.

The National Health Service Hospital Episode Statistics (HES) is a national data set detailing all completed consultant episodes in England. This includes emergency care where children are admitted to hospital. The data set does not capture details of children who are treated exclusively in the community, or for whom a fracture is identified in the Emergency Department and then managed in a fracture clinic. This is therefore an ideal data set to evaluate the volume of children admitted to hospitals in England as children with fractures are almost exclusively treated within the NHS.

The aim of this study is to evaluate the trends of children admitted to English hospitals since 2012. To do this annual incidence of fractures will be calculated and analysed to identify current trends and by evaluating the trends in operative management of paediatric fractures during this period.

Confidential: For Review Only

Methods

Research ethics approval was not required for this study as it was a secondary analysis of publicly accessible data.

Open access records for finished consultant episodes were retrieved from NHS Digital from 2012-2020 on the 20/02/2021 (https://digital.nhs.uk). Each consultant episode is coded by independent hospital coders with diagnoses from the International Classification of Diseases, 10th revision (ICD-10) classification system and interventions were coded using the Office of Population Services and Censuses, 5th edition (OPCS-4) code book[13 14].

The codes for acute fractures were classified into body regions (Upper limb: clavicle, scapula, humerus, forearm, hand. Lower limb: pelvis, hip, femur, patella, lower leg and foot. Spine: cervical spine, thoracic spine, lumbar spine and sacral spine. Chest: sternum or ribs). Primary procedures were identified and classified according to type of reduction and fixation method. Patients without a primary diagnosis of fracture were excluded to minimise duplications. Details on the codes included for each body region and procedure classification are supplied in supplementary materials.

The annual injury burden was calculated using the appropriate population estimate for different ages of children from the Office of National Statistics NOMIS service (https://www.nomisweb.co.uk/).

Incidence rates were calculated per 100,000 person years (PY) for all children and subdivided into four age groups (preschool 0-4, young children 5-9, older children 10-15, and adolescence 16-18). Annual trends were in incidence were visualised in Graphpad 7.04. Trends in injury incidence were fitted to a linear regression model with a significance level of <0.05.

Patient involvement

Patient involvement was not deemed to be appropriate for the design or delivery of this study as an analysis of open-source data.

Confidential: For Review Only

Results

The population of children living in England has increased from 12,094,205 in 2012 to 12,642,441 in 2019. The trends in population change were not uniform across all age groups. There has been an increase in the population of children aged 5-9 and 10-15 years, a decrease in the population of children aged 16-18 and no significant change in the population of children aged 0-4 (**Error! Reference source not found.**).

During the study period, 368,120 admissions were recorded with a primary diagnosis of a fracture. These included 256,008 upper limb fractures, 85,737 lower limb fractures, 20,939 skull or facial fractures, 4,542 spinal fractures and 894 chest fractures. The breakdown of these fractures into body area is shown in **Error! Reference source not found.**. Across all age groups, upper limb fractures were the most frequent fracture type accounting for 26.2-43.6% of fractures.

Fracture incidence was calculated per 100,000 person years. The trends for upper limb, lower limb, skull and facial fractures and spinal fractures are shown in **Error! Reference source not found.**. Skull and facial fractures in children aged 0-4 were demonstrated to be increasing during the study window with an annual increase of 0.629 per 100,000 PY (95% CI 0.367-0.891 p=0.0011). The incidence was found to be decreasing for five groups of fractures. These are: 1) upper limb fractures in adolescents (annual decrease of 3.49 per 100,000 PY, 95% CI 1.944-5.036 p=0.0015). 2) Lower limb fractures in preschool children (annual decrease 1.862 per 100,000 PY, 95% CI 1.222-2.502 p=0.0004). 3) lower limb fractures in younger children (annual decrease 1.773 per 100,000 PY, 95% CI 1.249-2.296 p=0.0002). 4) skull and facial fractures in adolescents (annual decrease 0.567 per 100,000 PY, 95% CI 0.208-0.926 p=0.0083) and 5) skull fractures in adolescents (annual decrease 1.03 per 100,000 PY 95% CI 0.026-2.034 p=0.046). There was insufficient evidence to demonstrate an increase or decrease in incidence rates for other fracture types.

The incidence of different fractures is broken down by specific body region in **Error! Reference source not found.**. In all age groups, the highest incidence of fractures was experienced in the upper limb with the forearm contributing the largest proportion of fractures in children aged 0-15. The peak incidence of forearm and humeral fractures was found in younger children while hand fractures were more common in older children. The lowest mean incidence of lower limb fractures was in younger children (46.7 per 100,000PY) Femoral fractures were the most common lower limb fracture in preschool children, whereas tibial and ankle fractures were most common all other age groups. The incidence of hip fractures is highest in older children and pelvic fractures highest in adolescents

Spine fractures were rare in preschool and younger children with a mean incidence of 0.4 per 100,000 PY for children aged 0-4 and 0.9 per 100,000 PY for children aged 5-9. There was a similar number of thoracic and lumber spine fractures. The highest incidence of cervical spine fractures was found in adolescents with a rate of 16.7 (95% CI 16.1-17.3) per 100,000 PY. This was higher than the combined rate of hip and femoral fractures.

The incidence of skull and facial bone fractures was lowest in younger children with a mean incidence of 7.5 per 100,000PY for children aged 5-9. The highest incidence was for adolescents at 50.1 per 100,000 PY. Chest fractures (rib or sternum) were rare through all age groups with a maximum mean incidence of 2.3 per 100,000PY for adolescents.

The trends in the rates for surgical management of childhood fractures and head injuries is shown in **Error! Reference source not found.**. There was a peak of manipulative management of fractures seen between 2013-2016 in children aged 5-15 which mirrors the peak in upper limb fracture

Confidential: For Review Only

incidence identified in the same time period. Linear regression analysis identified a statistically significant decrease in the rates of open reduction and fixation in younger children (annual decrease of 2.3 per 100,000 PY 95% CI 1.6-2.9 p=0.0002) and adolescents (annual decrease 2.5 per 100,000 PY, 95% CI 1.5-3.6 p=0.0010) and in closed reduction +/- fixation of older children (annual decrease 6.6 per 100,000PY, 95% CI 0.2-13.0 p=0.0446) and adolescents (annual decrease 2.5, 95% CI 1.5-3.6 p=0.0010). A decrease in skull or facial fracture surgery was demonstrated for younger children with an annual decrease of 0.13 (95% CI 0.0-0.2) per 100,000 PY and for adolescents with an annual decrease of -1.2 (95% CI 0.8-1.6) per 100,000 PY. No change in operative rates were found for spinal surgery in any age group or skull and facial surgery in preschool children.

Confidential: For Review Only

Discussion

There is very little contemporary literature describing the trends of hospital admissions due to childhood fractures. This study has demonstrated trends in fracture patterns, distributions and treatment strategies that are valuable for exploration in future research and in planning healthcare prioritisation.

While the dataset used has several advantages, including the national coverage of all NHS funded admissions and procedures, the most significant limitation is a lack of granularity within this dataset beyond number of finished consultant episodes and numbers of procedures. The data does not extend to the community rates of fractures, or for fractures treated exclusively in the Emergency Department. However, the does demonstrate a constant trend for most fractures, with a marginal decreasing trend in upper limb fractures in children aged 16-18, lower limb fractures in children aged 0-9, and skull fractures in children aged 10-18.

It is not clear what has been driving this decrease in limb and skull fractures in these groups. It is possible that national safety campaigns are reducing the injury burden on hospitals. The Department of Transport reported that 2019 had the lowest number of child and young adult casualties and fatalities following road traffic accidents which may be associated with changing demographics of road users and the influence of safety campaigns including "Think!"[15 16]. This may also be associated with an increase in sedentary lifestyles, though more work would be required to confirm this. Unfortunately, in the same time period, it has been identified that there has been a steady rise in penetrating trauma caused by knife crime, which with childhood admissions for knife wounds at the highest point in 2019 compared to 2012[17].

The trend in upper limb fractures did not show a significant change. However, inspection of the graph shows that there was a peak in the number of admissions 2014-2015 which corresponds to a peak in the number of closed reductions. There has been an increasing awareness of non-surgical management of upper limb fractures and Emergency Department manipulation which avoid inpatient admission[18-20].

The mean incidence of hospitalisation for childhood fractures was 374.8 per 100,000 children. This is lower than the 623.3 per 100,000 reported from Australia in the preceding decade (2002-2012) [21]. Part of this discrepancy may be that this previous study included admissions for nasal bone fractures, which contributed to 30% of the included facial fractures. The study from Australia also shows a decline in the number of admissions per 100,000 during the 10 years surveyed, which may have continued into the next decade[21]. We have found a higher proportion of lower limb fractures admitted to hospitals in England than in the Australian cohort. This may be due to an increasing trend in the U.K. of management of many upper limb fractures in the community.

General Practice databases have been used to develop estimates for fracture incidence in the United Kingdom. The Health Improvement Network (THIN) research database from 495 of the 8,228 general practitioners in the United Kingdom has been used to estimate the incidence of fractures in the under 5 population as 758 per 100,000 PY (95% confidence interval 748–769). Moon et al used the Clinical Practice Research Datalink (CPRD) database from 1988-2012 (which covers 6.9% of the UK population) to estimate a national incidence of 1,370 per 100,000 person years[22 23]. Given that we have not found any major changes in the trends of fracture admissions since 2012 then it seems that approximately 30% of U.K. fractures result in hospital admission, however the inpatient focus of the data for this study precludes a precise estimate.

Confidential: For Review Only

A marginal increase in the incidence of admissions for preschool head and facial was observed in the data. While mathematically significant, this represents increase of only 19 admissions per year in a preschool population of 3.3million. In this cohort, 83.5% of the fractures sustained by preschool children were to the base of skull or the skull vault with relatively few facial fractures. While this data set provides insufficient granularity to evaluate the mechanism of injury, it is possible that this increase may represent an increase in diagnosis of fractures following non-accidental injury. Robust evidence for trends in non-accidental injury is difficult to obtain, but a rise in non-accidental injury has been documented in the United States with 95% of children involved age <5[24 25]. Alternatively, there may be an increased awareness of the impact of serious head injuries in this age group driving this change.

This study has not included any hospital admissions from January 2020 onwards. The international coronavirus pandemic has been extensively documented to have caused significant disruption to Emergency care, with many papers identifying fewer children attending Emergency Departments for treatment following fractures during the pandemic[11 12 26-28]. This is likely to be due to lockdown policies limiting the availability of opportunities to participate in sport and adventurous play though in one series from London a reduction in non-surgical fractures was demonstrated without a corresponding decrease in surgical cases, suggesting that some more severe injuries were still occurring[29]. What is unclear is how the rates of injuries will respond to the relaxation of lockdown rules over the next 12-24 months and if the suppressed number of admissions will continue or if there will be a rebound back to (or exceeding) the rates identified in this current study.

Due to the nature of the study design, there remains some limitations. The use of finished consultant episodes has been used previously to estimate the number of admissions attributed to injuries and musculoskeletal pathology.[30 31] During the coding process, each patient episode is assigned up to 24 diagnostic codes. By analysing only primary diagnosis code it is possible that the estimates in this study may have undercounted where patients with multiple injuries have presented. However, this strategy has allowed us to present the primary reason for admission, accepting this limitation. As for all database studies, there will be an error rate associated with the input of data into the HES registry. However, the HES database is externally audited and validated by NHS Digital, with one hundred percent of finished consultant episodes being maintained with a primary diagnosis code in the included time period[32]. In this period, many of these confounders would be expected to occur at a constant rate, and while there may be an impact on the absolute values the trend results should not be grossly impacted.

Despite these limitations, this study has shown a decreasing trend for many childhood fractures in most age groups, except preschool skull fractures and adolescent spinal fractures, which are increasing. These trends can be used to benchmark service provision for anticipated hospital volume following easing of lockdown restrictions if childhood behaviour returns to 2018-2019 activities. There is also a need to ensure adequate provision of trained trauma staff in Emergency Departments to provide skilled manipulation to maintain and continue downward limb fracture admission trends. Additional work is required to evaluate the causes of the trends observed and to develop safety strategies to safeguard infants and adolescents from these injuries.

Confidential: For Review Only

Figures and tables

Table 1 Changes in demographics from the start and end of the study window. †Annual change calculated using a linear regression model

Age group	2012 population (% total)	2019 population (% total)	Annual change† (95% CI)	P value
**Preschool (0-4)**	3,393,356 (28.1%)	3,299,637 (26.1%)	-13533 (-27441 - 374)	0.055
**Younger children (5-9)**	3,083,582 (25.5%)	3,538,206 (28.0%)	66762 (52433 - 81092)	<0.0001
**Older children (10-15)**	3,653,288 (30.2%)	3,978,836 (31.5%)	49491 (20402 - 78580)	0.0059
**Adolescents (16-18)**	1,963,979 (16.2%)	1,825,762 (14.4%)	-20960 (-26060 - -15861)	<0.0001

Table 2 Distribution of fractures admitted to hospitals in England between 2012-2019 according to age of child. Numbers shown are absolute numbers of consultant episodes and proportion of total fracture load.

Body region	Preschool Age 0-4	Younger children Age 5-9	Older children Age 10-15	Adolescents Age 16-18
**Upper limb**	39,864 (32.7%)	93,648 (43.6%)	94,988 (34.8%)	27,508 (26.2%)
Forearm	*18,507 (15.2%)*	*60,950 (28.4%)*	*62,797 (23.0%)*	*8,576 (8.2%)*
Humerus	*14,820 (12.2%)*	*24,606 (11.5%)*	*8,600 (3.2%)*	*1,728 (1.6%)*
Hand	*3,809 (3.1%)*	*7,610 (3.5%)*	*20,940 (7.7%)*	*14,475 (13.8%)*
Clavicle	*2,678 (2.2%)*	*399 (0.2%)*	*2,403 (0.9%)*	*2,544 (2.4%)*
Scapula	*9 (0.0%)*	*9 (0.0%)*	*100 (0.0%)*	*126 (0.1%)*
Upper limb, unspecified	*9 (0.0%)*	*58 (0.0%)*	*95 (0.0%)*	*22(0.0%)*
Shoulder, unspecified	*32 (0.0%)*	*16 (0.0%)*	*53 (0.0%)*	*37(0.0%)*
**Lower limb**	17,292 (14.2%)	12,497 (5.8%)	37,622 (13.8%)	18,326 (17.4%)
Tibia/Fibula	*7,670 (6.3%)*	*8,088 (3.8%)*	*28,470 (0.4%)*	*12,501 (11.9%)*
Femur	*8,382 (6.9%)*	*2,804 (1.3%)*	*2,991 (1.1%)*	*1,856 (1.8%)*
Foot (not calcaneum or talus)	*613 (0.5%)*	*875 (0.4%)*	*2,418 (0.9%)*	*1,630 (1.6%)*
Hip	*522 (0.4%)*	*316 (0.1%)*	*1,244 (0.5%)*	*346 (0.3%)*
Pelvis	*56 (0.0%)*	*143 (0.1%)*	*936 (0.0%)*	*848 (0.8%)*
Patella	*13 (0.0%)*	*166 (0.1%)*	*1,126 (6.3%)*	*654 (0.6%)*
Talus	*10 (0.0%)*	*42 (0.0%)*	*239 (0.1%)*	*275 (0.3%)*
Calcaneum	*26 (0.0%)*	*63 (0.0%)*	*198 (0.1%)*	*216 (0.2%)*
**Spine**	112 (0.1%)	249 (0.1%)	1,451 (0.0%)	2,730 (2.6%)
Thoracic spine	*18 (0.0%)*	*106 (0.0%)*	*599 (0.2%)*	*911 (0.9%)*
Lumbar spine	*11 (0.0%)*	*69 (0.0%)*	*479 (0.2%)*	*1,058 (1.0%)*
Cervical spine	*80 (0.1%)*	*56 (0.0%)*	*294 (0.1%)*	*555 (0.5%)*
Sacral spine	*3 (0.0%)*	*18 (0.0%)*	*79 (0.0%)*	*206 (0.2%)*
**Skull and facial bones**	6,970 (5.7%)	2,025 (0.9%)	4,303 (1.6%)	7,641 (7.3%)
**Chest**	256 (0.2%)	64 (0.0%)	220 (0.1%)	354 (0.3%)

Confidential: For Review Only

Figure 1 Trends of fracture incidence for upper limb, lower limb, skull and facial fractures and spinal fractures between 2012-2019. Data presented separated by the age groups of children with trends and 95% confidence intervals calculated using a linear regression model.

Confidential: For Review Only

Table 3 Mean incidence of fractures per 100,000 person years (PY) admitted to hospitals in England between 2012-2019 according to age of child.

Body region	Preschool Age 0-4	Younger children Age 5-9	Older children Age 10-15	Adolescents Age 16-18
**Upper limb**	146.9	348.3	319.9	180.4
Forearm	*68.2*	*226.9*	*211.6*	*56.3*
Humerus	*54.6*	*91.3*	*28.9*	*11.3*
Hand	*14.0*	*28.3*	*70.5*	*94.9*
Clavicle	*9.9*	*1.5*	*8.1*	*16.7*
Scapula	*<0.1*	*<0.1*	*0/3*	*0.8*
Upper limb, unspecified	*<0.1*	*0.2*	*0.3*	*0.1*
Shoulder, unspecified	*0.1*	*0.1*	*0.2*	*0.2*
**Lower limb**	63.7	46.7	126.5	120.3
Tibia/Fibula	*28.2*	*30.2*	*95.7*	*82.1*
Femur	*30.9*	*10.4*	*10.1*	*12.2*
Foot (not calcaneum or talus)	*2.3*	*3.3*	*8.1*	*10.7*
Hip	*1.9*	*1.2*	*4.2*	*2.3*
Pelvis	*0.2*	*0.5*	*3.1*	*5.6*
Patella	*<0.1*	*0.6*	*3.8*	*4.3*
Talus	*<0.1*	*0.2*	*0.8*	*1.8*
Calcaneum	*0.1*	*0.2*	*0.7*	*1.4*
**Spine**	0.4	0.9	4.9	17.9
Thoracic spine	*0.1*	*0.4*	*2.0*	*6.0*
Lumbar spine	*<0.1*	*0.3*	*1.6*	*7.0*
Cervical spine	*0.3*	*0.2*	*1.0*	*16.7*
Sacral spine	*<0.1*	*0.1*	*0.3*	*1.4*
**Skull and facial bones**	25.7	7.5	14.5	50.1
**Chest**	0.9	0.2	0.7	2.3

Figure 2 Trends of fracture interventions for limb open or closed reductions, skull and facial fractures and spinal fractures between 2012-2019. Data presented separated by the age groups of children with trends and 95% confidence intervals calculated using a linear regression model.

Confidential: For Review Only

1. Landin LA. Fracture patterns in children. Analysis of 8,682 fractures with special reference to incidence, etiology and secular changes in a Swedish urban population 1950-1979. *Acta orthopaedica Scandinavica Supplementum* 1983;202:1-109. [published Online First: 1983/01/01]
2. Tiderius CJ, Landin L, Düppe H. Decreasing incidence of fractures in children: an epidemiological analysis of 1,673 fractures in Malmö, Sweden, 1993-1994. *Acta Orthop Scand* 1999;70(6):622-6. doi: 10.3109/17453679908997853 [published Online First: 2000/02/09]
3. Kang MS, Kim HS. Characteristics and trends of traumatic injuries in children visiting emergency departments in South Korea: A retrospective serial cross-sectional study using both nationwide-sample and single-institutional data. *PLoS One* 2019;14(8):e0220798. doi: 10.1371/journal.pone.0220798 [published Online First: 2019/08/10]
4. Bergman E, Lempesis V, Nilsson J-Å, et al. Time trends in pediatric fractures in a Swedish city from 1950 to 2016. *Acta Orthopaedica* 2020;91(5):598-604. doi: 10.1080/17453674.2020.1783484
5. Orces CH, Orces J. Trends in the U.S. Childhood Emergency Department Visits for Fall-Related Fractures, 2001-2015. *Cureus* 2020;12(11):e11629. doi: 10.7759/cureus.11629 [published Online First: 2020/12/31]
6. Ding R, McCarthy ML, Houseknecht E, et al. The health-related quality of life of children with an extremity fracture: a one-year follow-up study. *J Pediatr Orthop* 2006;26(2):157-63. doi: 10.1097/01.bpo.0000218521.98244.7e [published Online First: 2006/03/25]
7. Kendrick D, Vinogradova Y, Coupland C, et al. Recovery from injury: the UK burden of injury multicentre longitudinal study. *Injury prevention* 2013;19(6):370-81.
8. Schneeberg A, Ishikawa T, Kruse S, et al. A longitudinal study on quality of life after injury in children. *Health and quality of life outcomes* 2016;14(1):120.
9. FANZCA M. Distress at induction of anaesthesia in children. A survey of incidence, associated factors and recovery characteristics. *Pediatric Anesthesia* 1998;8(5):383-92.
10. Bentley J. Distress in children attending A&E. *Emergency nurse* 2004;12(4)
11. Murphy T, Akehurst H, Mutimer J. Impact of the 2020 COVID-19 pandemic on the workload of the orthopaedic service in a busy UK district general hospital. *Injury* 2020;51(10):2142-47.
12. Sugand K, Park C, Morgan C, et al. Impact of the COVID-19 pandemic on paediatric orthopaedic trauma workload in central London: a multi-centre longitudinal observational study over the "golden weeks". *Acta Orthop* 2020;91(6):633-38. doi: 10.1080/17453674.2020.1807092 [published Online First: 2020/08/25]
13. Digital N. OPCS Classification of Interventions and Procedures 2021 [Available from: https://datadictionary.nhs.uk/supporting_information/opcs_classification_of_interventions_and_procedures.html accessed 30/4/2021.
14. Organization WH. ICD-10 : international statistical classification of diseases and related health problems : tenth revision. 2nd ed. Geneva: World Health Organization. , 2004.
15. Murphy A. Reported road casualties in Great Britain: 2019 annual report. London: Department for Transport, 2020.
16. Transport Df. Think! 2021 [Available from: https://www.think.gov.uk/ accessed 30/4/2021.
17. Allen G, Kirk-Wade E. Knife crime in England and Wales. Briefing Paper. London: House of Commons Library, 2020.
18. Kurien T, Price K, Pearson RG, et al. Manipulation and reduction of paediatric fractures of the distal radius and forearm using intranasal diamorphine and 50% oxygen and nitrous oxide in the emergency department: a 2.5-year study. *The bone & joint journal* 2016;98(1):131-36.
19. Marson BA, Ng JW, Craxford S, et al. Treatment of completely displaced distal radial fractures with a straight plaster or manipulation under anaesthesia. *The Bone & Joint Journal* 2021:1-6.
20. Patel D, Talbot C, Luo W, et al. The use of esketamine sedation in the emergency department for manipulation of paediatric forearm fractures: A 5 year study. *Injury* 2021

Confidential: For Review Only

21. Faris M, Lystad RP, Harris I, et al. Fracture-related hospitalisations and readmissions of Australian children ≤16 years: A 10-year population-based cohort study. *Injury* 2020;51(10):2172-78. doi: 10.1016/j.injury.2020.07.032 [published Online First: 2020/07/28]
22. Moon RJ, Harvey NC, Curtis EM, et al. Ethnic and geographic variations in the epidemiology of childhood fractures in the United Kingdom. *Bone* 2016;85:9-14. doi: 10.1016/j.bone.2016.01.015 [published Online First: 2016/01/24]
23. Orton E, Kendrick D, West J, et al. Persistence of health inequalities in childhood injury in the UK: a population-based cohort study of children under 5. *PLoS One* 2014;9(10):e111631. doi: 10.1371/journal.pone.0111631 [published Online First: 2014/10/28]
24. Rosenfeld EH, Johnson B, Wesson DE, et al. Understanding non-accidental trauma in the United States: A national trauma databank study. *Journal of Pediatric Surgery* 2020;55(4):693-97. doi: https://doi.org/10.1016/j.jpedsurg.2019.03.024
25. Thompson LW, Bass K, Agyei J, et al. Rising Incidence of Non-Accidental Trauma in Infants: A Call to Revisit Prevention Strategies. *Pediatrics* 2018;142:749-49. doi: 10.1542/peds.142.1_MeetingAbstract.749
26. Bram JT, Johnson MA, Magee LC, et al. Where have all the fractures gone? The epidemiology of pediatric fractures during the COVID-19 pandemic. *Journal of Pediatric Orthopaedics* 2020;40(8):373-79.
27. Nabian MH, Vosoughi F, Najafi F, et al. Epidemiological pattern of pediatric trauma in COVID-19 outbreak: Data from a tertiary trauma center in Iran. *Injury* 2020;51(12):2811-15. doi: 10.1016/j.injury.2020.09.015 [published Online First: 2020/09/23]
28. Pines JM, Zocchi MS, Black BS, et al. Characterizing pediatric emergency department visits during the COVID-19 pandemic. *The American journal of emergency medicine* 2021;41:201-04.
29. Ibrahim Y, Huq S, Shanmuganathan K, et al. Trampolines injuries are bouncing back: The effect of the COVID-19 UK lockdown on the paediatric trauma burden. *Bone & Joint Open* 2021;2(2):86.
30. Judge A, Murphy R, Maxwell R, et al. Temporal trends and geographical variation in the use of subacromial decompression and rotator cuff repair of the shoulder in England. *The bone & joint journal* 2014;96(1):70-74.
31. Manley OW, Wormald JC, Furniss D. The changing shape of hand trauma: an analysis of Hospital Episode Statistics in England. *Journal of hand surgery (European volume)* 2019;44(5):532-36.
32. Digital N. HES Data Quality Statement 2020 [Available from: https://digital.nhs.uk/data-and-information/publications/statistical/hospital-admitted-patient-care-activity/2019-20/data-quality-statement accessed 30/4/2021.

Confidential: For Review Only

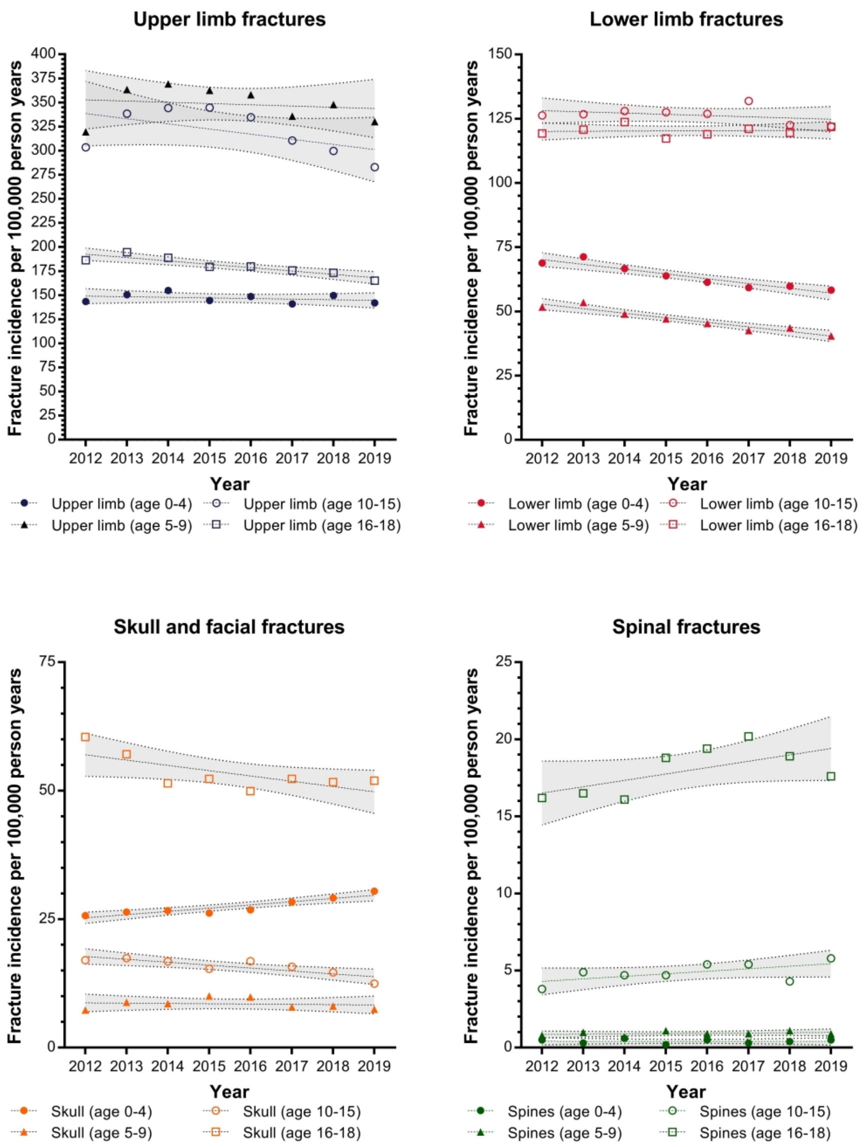

200x253mm (600 x 600 DPI)

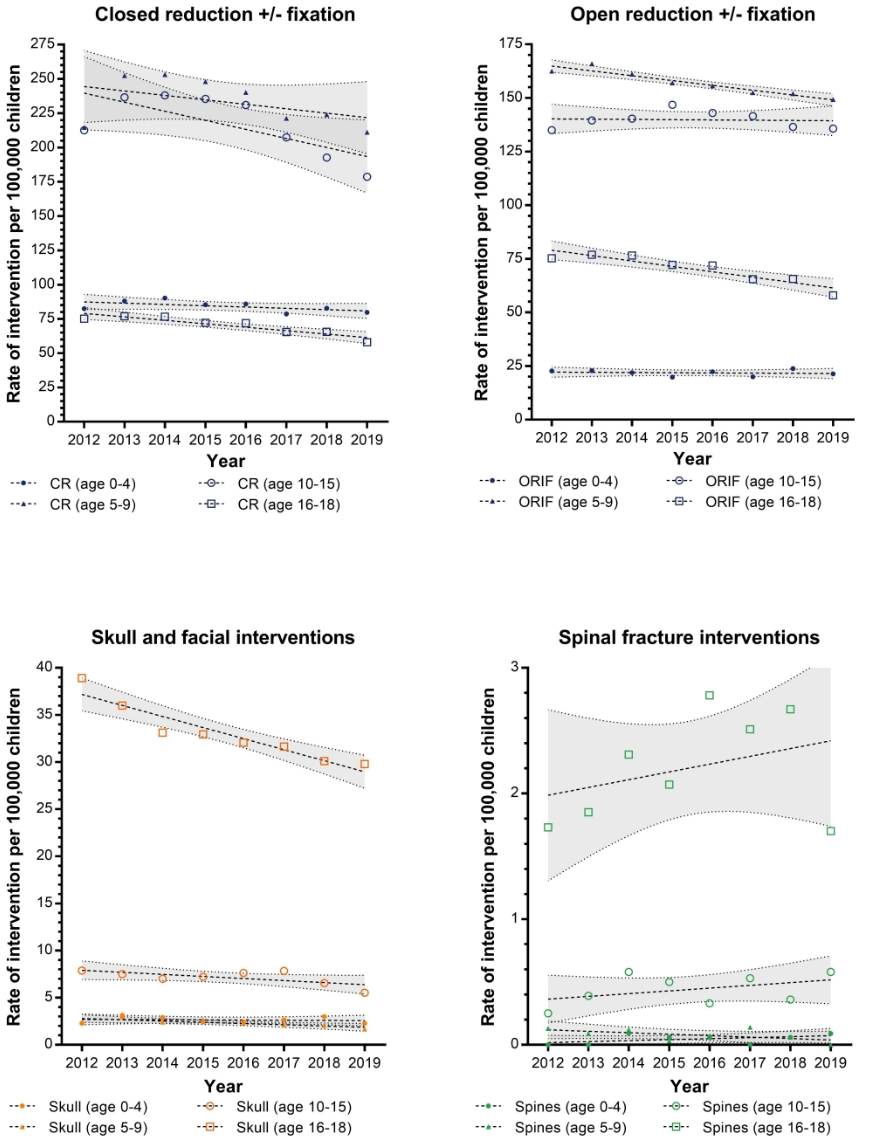

195x247mm (600 x 600 DPI)

List of included codes

code	Name	Mapped to body area
S12.0	Fracture of first cervical vertebra	C-spine
S12.1	Fracture of second cervical vertebra	C-spine
S12.2	Fracture of other specified cervical vertebra	C-spine
S12.8	Fracture of other parts of neck	C-spine
S12.9	Fracture of neck, part unspecified	C-spine
S62.0	Fracture of navicular [scaphoid] bone of han	carpus
S62.1	Fracture of other carpal bone(s)	carpus
P13.4	Fracture of clavicle due to birth injury	clavicle
S42.0	Fracture of clavicle	clavicle
S72.3	Fracture of shaft of femur	femur
S72.4	Fracture of lower end of femur	femur
S72.8	Fractures of other parts of femur	femur
S72.9	Fracture of femur, part unspecified	femur
S92.9	Fracture of foot, unspecified	foot
S52.0	Fracture of upper end of ulna	Forearm
S52.1	Fracture of upper end of radius	Forearm
S52.2	Fracture of shaft of ulna	Forearm
S52.3	Fracture of shaft of radius	Forearm
S52.4	Fracture of shafts of both ulna and radius	Forearm
S52.5	Fracture of lower end of radius	Forearm
S52.6	Fracture of lower end of both ulna and radiu	Forearm
S52.8	Fracture of other parts of forearm	Forearm
S52.9	Fracture of forearm, part unspecified	Forearm
S62.5	Fracture of thumb	hand
S62.6	Fracture of other finger	hand
S62.8	Fracture of other and unspecified parts of wr	hand
S92.0	Fracture of calcaneus	hindfoot
S92.1	Fracture of talus	hindfoot
S72.0	Fracture of neck of femur	hip
S72.1	Pertrochanteric fracture	hip
S72.2	Subtrochanteric fracture	hip
S42.2	Fracture of upper end of humerus	humerus
S42.3	Fracture of shaft of humerus	humerus
S42.4	Fracture of lower end of humerus	humerus
S32.0	Fracture of lumbar vertebra	L-spine
S82.1	Fracture of upper end of tibia	lower leg
S82.2	Fracture of shaft of tibia	lower leg
S82.3	Fracture of lower end of tibia	lower leg
S82.4	Fracture of fibula alone	lower leg
S82.5	Fracture of medial malleolus	lower leg
S82.6	Fracture of lateral malleolus	lower leg
S82.8	Fractures of other parts of lower leg	lower leg
S82.9	Fracture of lower leg, part unspecified	lower leg
S62.2	Fracture of first metacarpal bone	metacarp
S62.3	Fracture of other metacarpal bone	metacarp
S82.0	Fracture of patella	patella
S32.1	Fracture of sacrum	pelvis
S32.2	Fracture of coccyx	pelvis
S32.3	Fracture of ilium	pelvis
S32.4	Fracture of acetabulum	pelvis
S32.5	Fracture of pubis	pelvis
S32.8	Fracture of other and unspecified parts of lu	pelvis
S42.1	Fracture of scapula	scapula
P13.0	Fracture of skull due to birth injury	skull
S02.0	Fracture of vault of skull	skull
S02.1	Fracture of base of skull	skull
S02.3	Fracture of orbital floor	skull
S02.4	Fracture of malar and maxillary bones	skull
S02.6	Fracture of mandible	skull
S02.8	Fractures of other skull and facial bones	skull
S02.9	Fracture of skull and facial bones, part unsp	skull

Confidential: For Review Only

T08.X	Fracture of spine, level unspecified	spine
S22.0	Fracture of thoracic vertebra	t0spine
S92.2	Fracture of other tarsal bone(s)	tarsal
S92.3	Fracture of metatarsal bone	tarsal
S22.2	Fracture of sternum	thorax
S22.3	Fracture of rib	thorax
S22.8	Fracture of other parts of bony thorax	thorax
S22.9	Fracture of bony thorax, part unspecified	thorax
S92.4	Fracture of great toe	toe
S92.5	Fracture of other toe	toe

Confidential: For Review Only

BMJ Paediatrics Open

Trends in hospital admissions for childhood fractures in England

Journal:	*BMJ Paediatrics Open*
Manuscript ID	bmjpo-2021-001187.R2
Article Type:	Original research
Date Submitted by the Author:	11-Oct-2021
Complete List of Authors:	Marson, Ben; University of Nottingham School of Medicine, Orthopaedics and Trauma Group<br>Manning, Joseph ; Nottingham University Hospitals NHS Trust, Nottingham Children's Hospital; University of Nottingham, Children and Young people Health Research, School of Health Sciences<br>James, Marilyn; University of Nottingham School of Medicine, Clinical Trials Unit<br>Ikram, Adeel; University of Nottingham School of Medicine, Orthopaedics and Trauma Group<br>Bryson, David; Nottingham University Hospitals NHS Trust, Nottingham Children's Hospital<br>Ollivere, Benjamin; University of Nottingham School of Medicine, Orthopaedics and Trauma Group
Keywords:	Epidemiology

SCHOLARONE™
Manuscripts

I, the Submitting Author has the right to grant and does grant on behalf of all authors of the Work (as defined in the below author licence), an exclusive licence and/or a non-exclusive licence for contributions from authors who are: i) UK Crown employees; ii) where BMJ has agreed a CC-BY licence shall apply, and/or iii) in accordance with the terms applicable for US Federal Government officers or employees acting as part of their official duties; on a worldwide, perpetual, irrevocable, royalty-free basis to BMJ Publishing Group Ltd ("BMJ") its licensees and where the relevant Journal is co-owned by BMJ to the co-owners of the Journal, to publish the Work in this journal and any other BMJ products and to exploit all rights, as set out in our licence.

The Submitting Author accepts and understands that any supply made under these terms is made by BMJ to the Submitting Author unless you are acting as an employee on behalf of your employer or a postgraduate student of an affiliated institution which is paying any applicable article publishing charge ("APC") for Open Access articles. Where the Submitting Author wishes to make the Work available on an Open Access basis (and intends to pay the relevant APC), the terms of reuse of such Open Access shall be governed by a Creative Commons licence – details of these licences and which Creative Commons licence will apply to this Work are set out in our licence referred to above.

Other than as permitted in any relevant BMJ Author's Self Archiving Policies, I confirm this Work has not been accepted for publication elsewhere, is not being considered for publication elsewhere and does not duplicate material already published. I confirm all authors consent to publication of this Work and authorise the granting of this licence.

Confidential: For Review Only

Trends in hospital admissions for childhood fractures in England

Word count (2518 words)

Marson BA, NIHR Doctoral Fellow, Department of Trauma and Orthopaedics, University of Nottingham NG7 2UH. Ben.marson@nottingham.ac.uk

Manning JC, Associate Professor, School of Health Sciences, University of Nottingham, NG7 2UH

James M Professor of Health Economics Faculty of Medicine & Health Sciences, University of Nottingham, NG7 2UH

Ikram A, Doctoral Fellow, Department of Trauma and Orthopaedics, University of Nottingham NG7 2UH)

Bryson DJ, Consultant in Paediatric Orthopaedics. Nottingham University Hospitals NHS trust, QMC, Nottingham, NG7 2UH

Ollivere BJ, Professor of Trauma Department of Trauma and Orthopaedics, University of Nottingham NG7 2UH

Confidential: For Review Only

The Corresponding Author has the right to grant on behalf of all authors and does grant on behalf of all authors, an exclusive licence (or non exclusive for government employees) on a worldwide basis to the BMJ Publishing Group Ltd to permit this article (if accepted) to be published in BMJ editions and any other BMJPGL products and sublicences such use and exploit all subsidiary rights, as set out in our licence.

The manuscript guarantor (BJO) affirms that this manuscript is an honest, accurate, and transparent account of the study being reported; that no important aspects of the study have been omitted; and that any discrepancies from the study as planned (and, if relevant, registered) have been explained.

All authors have completed the ICMJE uniform disclosure at www.icmje.org/coi_disclosure.pdf and declare: financial support for the submitted work from NIHR; BAM has received a research grant from the NIHR to support this work. JM is funded through a NIHR grant unrelated to this work. Other authors have no support from any organisation for the submitted work; no financial relationships with any organisations that might have an interest in the submitted work in the previous three years; no other relationships or activities that could appear to have influenced the submitted work.

Contributorship statement

BAM: Data acquisition, analysis, writing of first draft

JCM: Study conceptualisation, analysis, editing of final draft, supervision of trainee

MJ: Study conceptualisation, analysis, editing of final draft, supervision of trainee

AI: Data acquisition, analysis, editing of final draft

DJB: Study conceptualisation, analysis, contextualisation of study, editing of final draft, supervision of trainees

BJO: Study conceptualisation, analysis, editing of final draft, supervision of trainees

Abstract

Purpose

Fractures to the axial and appendicular skeleton are common in children causing loss of opportunities and disability. There are relatively few studies available to quantify the number of children who have their fractures diagnosed in the Emergency Department and are then admitted to hospital for ongoing management. The purpose of this study is to explore trends of frequency, types and age of children sustaining fractures who were admitted for intervention to NHS hospitals.

Design

The study uses data from the Hospital Episode Statistics and Office for National Statistics from 2012-2019 to calculate the annual incidence of hospital admission for limb, spine, facial and skull fractures per 100,000 children.

Results

During 2012-2019, 368,120 children were admitted to English NHS hospitals with a fracture. 256,018 (69.5%) were upper limb, 85,737 (23.3%) were lower limb fractures and 20,939 (5.7%) were skull or facial fractures. The annual incidence of upper limb fractures was highest in children aged 5-9 (348.3 per 100,000 children) and the highest incidence of lower limb fractures was in children aged 10-15(126.5 per 100,000 children). The incidence of skull and facial fractures in preschool (age 0-4) children has been increasing at a rate of 0.629 per 100,000 children per year.

Implications

The annual incidence of hospital admission for fractures in children has been shown to be consistent for several fracture types between 2012-2019. An increasing trend of admissions with preschool skull fractures was observed, though the study data does not have sufficient granularity to demonstrate if this is due to changes in practice or to accidental or non-accidental causes.

What is already known on the topic.

1. Fractures in children are common with more hospital admissions for upper limb than lower limb fractures.
2. Previous estimates of hospital admissions following childhood fractures are limited by a lack of robust denominator.

What this study adds

1. Contemporary estimates of incidence for hospital admission for limb, chest, spinal and skull and facial fractures.
2. The peak of upper limb fracture incidence is in 5–9 year-old children and for lower limb fractures it is in 10-15 year olds.
3. Demonstration of stable or gradually reducing trends of admissions for most fractures except preschool skull fractures.

Confidential: For Review Only

Introduction

Fractures in children are a common presentation to the Emergency Department, with overall estimated annual incidence rates of 1,500-3,600 fractures per 100,000 children per year[1-5]. Fractures in childhood cause pain and loss of opportunity[6-8]. Most children contained within these series are managed in the community. However, some children require hospital admission and treatment. Admission to hospital is distressing, which is amplified if the child requires surgery[9 10], yet there is relatively little information regarding the numbers of children admitted for hospital treatment of fractures.

There have been several reports of decreased numbers of children presenting to hospitals with fractures during lockdown restrictions due to the coronavirus pandemic[11 12]. These studies typically report absolute numbers of children and are not able to calculate rates of fractures due to uncertainty regarding the population size or denominator number. An evaluation of injury burden and trends in a population with a known denominator is important to allow for optimal service planning both in anticipation of removal of restrictions and for future years.

The National Health Service Hospital Episode Statistics (HES) is a national data set detailing all completed consultant episodes in England. This includes emergency care where children are admitted to hospital. The data set does not capture details of children who are treated exclusively in the community, or for whom a fracture is identified in the Emergency Department and then managed in a fracture clinic. This is therefore an ideal data set to evaluate the volume of children admitted to hospitals in England as children with fractures are almost exclusively treated within the NHS.

The aim of this study is to evaluate the trends of children admitted to English hospitals since 2012. To do this annual incidence of fractures will be calculated and analysed to identify current trends and by evaluating the trends in operative management of paediatric fractures during this period.

Confidential: For Review Only

Methods

Research ethics approval was not required for this study as it was a secondary analysis of publicly accessible data.

Open access records for finished consultant episodes were retrieved from NHS Digital from 2012-2020 on the 20/02/2021 (https://digital.nhs.uk). Each consultant episode is coded by independent hospital coders with diagnoses from the International Classification of Diseases, 10th revision (ICD-10) classification system and interventions were coded using the Office of Population Services and Censuses, 5th edition (OPCS-4) code book[13 14].

The codes for acute fractures were classified into body regions (Upper limb: clavicle, scapula, humerus, forearm, hand. Lower limb: pelvis, hip, femur, patella, lower leg and foot. Spine: cervical spine, thoracic spine, lumbar spine and sacral spine. Chest: sternum or ribs). Primary procedures were identified and classified according to type of reduction and fixation method (See Supplementary File 1). Patients without a primary diagnosis of fracture were excluded to minimise duplications.

The annual injury burden was calculated using the appropriate population estimate for different ages of children from the Office of National Statistics NOMIS service (https://www.nomisweb.co.uk/).

Incidence rates were calculated per 100,000 person years (PY) for all children and subdivided into four age groups (preschool 0-4, young children 5-9, older children 10-15, and adolescence 16-18). Annual trends were in incidence were visualised in Graphpad 7.04. Trends in injury incidence were fitted to a linear regression model with a significance level of <0.05.

Patient involvement

Patient involvement was not deemed to be appropriate for the design or delivery of this study as an analysis of open-source data.

Confidential: For Review Only

Results

The population of children living in England has increased from 12,094,205 in 2012 to 12,642,441 in 2019. The trends in population change were not uniform across all age groups. There has been an increase in the population of children aged 5-9 and 10-15 years, a decrease in the population of children aged 16-18 and no significant change in the population of children aged 0-4 (Table 1).

During the study period, 368,120 admissions were recorded with a primary diagnosis of a fracture. These included 256,008 upper limb fractures, 85,737 lower limb fractures, 20,939 skull or facial fractures, 4,542 spinal fractures and 894 chest fractures. The breakdown of these fractures into body area is shown in Table 2. Across all age groups, upper limb fractures were the most frequent fracture type accounting for 26.2-43.6% of fractures.

Fracture incidence was calculated per 100,000 person years. The trends for upper limb, lower limb, skull and facial fractures and spinal fractures are shown in Figure 1. Skull and facial fractures in children aged 0-4 were demonstrated to be increasing during the study window with an annual increase of 0.629 per 100,000 PY (95% CI 0.367-0.891 p=0.0011). The incidence was found to be decreasing for five groups of fractures. These are: 1) upper limb fractures in adolescents (annual decrease of 3.49 per 100,000 PY, 95% CI 1.944-5.036 p=0.0015). 2) Lower limb fractures in preschool children (annual decrease 1.862 per 100,000 PY, 95% CI 1.222-2.502 p=0.0004). 3) lower limb fractures in younger children (annual decrease 1.773 per 100,000 PY, 95% CI 1.249-2.296 p=0.0002). 4) skull and facial fractures in adolescents (annual decrease 0.567 per 100,000 PY, 95% CI 0.208-0.926 p=0.0083) and 5) skull fractures in adolescents (annual decrease 1.03 per 100,000 PY 95% CI 0.026-2.034 p=0.046). There was insufficient evidence to demonstrate an increase or decrease in incidence rates for other fracture types.

The incidence of different fractures is broken down by specific body region in Table 3. In all age groups, the highest incidence of fractures was experienced in the upper limb with the forearm contributing the largest proportion of fractures in children aged 0-15. The peak incidence of forearm and humeral fractures was found in younger children while hand fractures were more common in older children. The lowest mean incidence of lower limb fractures was in younger children (46.7 per 100,000PY) Femoral fractures were the most common lower limb fracture in preschool children, whereas tibial and ankle fractures were most common all other age groups. The incidence of hip fractures is highest in older children and pelvic fractures highest in adolescents

Spine fractures were rare in preschool and younger children with a mean incidence of 0.4 per 100,000 PY for children aged 0-4 and 0.9 per 100,000 PY for children aged 5-9. There was a similar number of thoracic and lumber spine fractures. The highest incidence of cervical spine fractures was found in adolescents with a rate of 16.7 (95% CI 16.1-17.3) per 100,000 PY. This was higher than the combined rate of hip and femoral fractures.

The incidence of admissions for skull and facial bone fractures was lowest in younger children with a mean incidence of 7.5 per 100,000PY for children aged 5-9. The highest incidence was for adolescents at 50.1 per 100,000 PY. Chest fractures (rib or sternum) were rare through all age groups with a maximum mean incidence of 2.3 per 100,000PY for adolescents.

The trends in the rates for surgical management of childhood fractures and head injuries is shown in Figure 2. There was a peak of manipulative management of fractures seen between 2013-2016 in children aged 5-15 which mirrors the peak in admissions for upper limb fracture incidence identified in the same time period. Linear regression analysis identified a statistically significant decrease in the rates of open reduction and fixation in younger children (annual decrease of 2.3 per 100,000 PY 95%

Confidential: For Review Only

CI 1.6-2.9 p=0.0002) and adolescents (annual decrease 2.5 per 100,000 PY, 95% CI 1.5-3.6 p=0.0010) and in closed reduction +/- fixation of older children (annual decrease 6.6 per 100,000PY, 95% CI 0.2-13.0 p=0.0446) and adolescents (annual decrease 2.5, 95% CI 1.5-3.6 p=0.0010). A decrease in skull or facial fracture surgery was demonstrated for younger children with an annual decrease of 0.13 (95% CI 0.0-0.2) per 100,000 PY and for adolescents with an annual decrease of -1.2 (95% CI 0.8-1.6) per 100,000 PY. No change in operative rates were found for spinal surgery in any age group or skull and facial surgery in preschool children.

Confidential: For Review Only

Discussion

There is very little contemporary literature describing the trends of hospital admissions due to childhood fractures. This study has demonstrated trends in fracture patterns, distributions and treatment strategies that are valuable for exploration in future research and in planning healthcare prioritisation.

While the dataset used has several advantages, including the national coverage of all NHS funded admissions and procedures, the most significant limitation is a lack of granularity within this dataset beyond number of finished consultant episodes and numbers of procedures. The data does not extend to the community rates of fractures, or for fractures treated exclusively in the Emergency Department. However, they demonstrate a constant trend for most fractures, with a marginal decreasing trend in admissions for upper limb fractures in children aged 16-18, lower limb fractures in children aged 0-9, and skull fractures in children aged 10-18.

It is not clear what has been driving this decrease in limb and skull fracture admissions in these groups. It is possible that national safety campaigns are reducing the injury burden on hospitals. The Department of Transport reported that 2019 had the lowest number of child and young adult casualties and fatalities following road traffic accidents which may be associated with changing demographics of road users and the influence of safety campaigns including “Think!”[15 16]. This may also be associated with an increase in sedentary lifestyles, though more work would be required to confirm this. Unfortunately, in the same time period, it has been identified that there has been a steady rise in penetrating trauma caused by knife crime, which with childhood admissions for knife wounds at the highest point in 2019 compared to 2012[17].

The trend in upper limb fractures did not show a significant change. However, inspection of the graph shows that there was a peak in the number of admissions 2014-2015 which corresponds to a peak in the number of closed reductions. There has been an increasing awareness of non-surgical management of limb fractures and Emergency Department manipulation which avoid inpatient admission[18-22]. Importantly, such fractures and their management would not be captured in this data.

The mean incidence of hospitalisation for childhood fractures was 374.8 per 100,000 children. This is lower than the 623.3 per 100,000 reported from Australia in the preceding decade (2002-2012) [23]. Part of this discrepancy may be that this previous study included admissions for nasal bone fractures, which contributed to 30% of the included facial fractures. The study from Australia also shows a decline in the number of admissions per 100,000 during the 10 years surveyed, which may have continued into the next decade[23]. We have found a higher proportion of lower limb fractures admitted to hospitals in England than in the Australian cohort. This may be due to an increasing trend in the U.K. of management of many upper limb fractures in the community.

General Practice databases have been used to develop estimates for fracture incidence in the United Kingdom. The Health Improvement Network (THIN) research database from 495 of the 8,228 general practitioners in the United Kingdom has been used to estimate the incidence of fractures in the under 5 population as 758 per 100,000 PY (95% confidence interval 748–769). Moon et al used the Clinical Practice Research Datalink (CPRD) database from 1988-2012 (which covers 6.9% of the UK population) to estimate a national incidence of 1,370 per 100,000 person years[24 25]. Given that we have not found any major changes in the trends of fracture admissions since 2012 then it seems that approximately 30% of U.K. fractures result in hospital admission, however the inpatient focus of the data for this study precludes a precise estimate.

Confidential: For Review Only

A marginal increase in the incidence of admissions for preschool head and facial fractures was observed in the data. While mathematically significant, this represents an increase of only 19 admissions per year in a preschool population of 3.3million. In this cohort, 83.5% of the fractures sustained by preschool children were to the base of skull or the skull vault with relatively few facial fractures. While this data set provides insufficient granularity to evaluate the mechanism of injury, it is possible that this increase may represent an increase in diagnosis of fractures following non-accidental injury. Also, it could reflect changes in patterns of investigations, e.g. skull CT, and management. Robust evidence for trends in non-accidental injury is difficult to obtain, but a rise in non-accidental injury has been documented in the United States with 95% of children involved age <5 years[26 27]. Alternatively, there may be an increased awareness of the impact of serious head injuries in this age group and thus a lower barrier to admissions driving this change.

This study has not included any hospital admissions from January 2020 onwards. The international coronavirus pandemic has been extensively documented to have caused significant disruption to Emergency care, with many papers identifying fewer children attending Emergency Departments for treatment following fractures during the pandemic[11 12 28-30]. This is likely to be due to lockdown policies limiting the availability of opportunities to participate in sport and adventurous play though in one series from London a reduction in non-surgical fractures was demonstrated without a corresponding decrease in surgical cases, suggesting that some more severe injuries were still occurring[31]. What is unclear is how the rates of injuries will respond to the relaxation of lockdown rules over the next 12-24 months and if the suppressed number of admissions will continue or if there will be a rebound back to (or exceeding) the rates identified in this current study.

Due to the nature of the study design, there remains some limitations. The use of finished consultant episodes has been used previously to estimate the number of admissions attributed to injuries and musculoskeletal pathology.[32 33] During the coding process, each patient episode is assigned up to 24 diagnostic codes. By analysing only primary diagnosis code it is possible that the estimates in this study may have undercounted where patients with multiple injuries have presented. However, this strategy has allowed us to present the primary reason for admission, accepting this limitation. As for all database studies, there will be an error rate associated with the input of data into the HES registry. However, the HES database is externally audited and validated by NHS Digital, with one hundred percent of finished consultant episodes being maintained with a primary diagnosis code in the included time period[34]. In this period, many of these confounders would be expected to occur at a constant rate, and while there may be an impact on the absolute values the trend results should not be grossly impacted.

Despite these limitations, this study has shown a decreasing trend of admissions for many childhood fractures in most age groups, except preschool skull fractures and adolescent spinal fractures, which are increasing. These trends can be used to benchmark service provision for anticipated hospital volume following easing of lockdown restrictions if childhood behaviour returns to 2018-2019 activities. There is also a need to ensure adequate provision of trained trauma staff in Emergency Departments to provide skilled manipulation to maintain and continue downward limb fracture admission trends. Additional work is required to evaluate the causes of the trends observed and to develop safety strategies to safeguard infants and adolescents from these injuries.

Confidential: For Review Only

Figures and tables

Table 1 Changes in demographics from the start and end of the study window. †Annual change calculated using a linear regression model

Age group	2012 population (% total)	2019 population (% total)	Annual change† (95% CI)	P value
**Preschool (0-4)**	3,393,356 (28.1%)	3,299,637 (26.1%)	-13533 (-27441 - 374)	0.055
**Younger children (5-9)**	3,083,582 (25.5%)	3,538,206 (28.0%)	66762 (52433 - 81092)	<0.0001
**Older children (10-15)**	3,653,288 (30.2%)	3,978,836 (31.5%)	49491 (20402 - 78580)	0.0059
**Adolescents (16-18)**	1,963,979 (16.2%)	1,825,762 (14.4%)	-20960 (-26060 - -15861)	<0.0001

Table 2 Distribution of fractures admitted to hospitals in England between 2012-2019 according to age of child. Numbers shown are absolute numbers of consultant episodes and proportion of total fracture load.

Body region	Preschool Age 0-4	Younger children Age 5-9	Older children Age 10-15	Adolescents Age 16-18
**Upper limb**	39,864 (32.7%)	93,648 (43.6%)	94,988 (34.8%)	27,508 (26.2%)
Forearm	*18,507 (15.2%)*	*60,950 (28.4%)*	*62,797 (23.0%)*	*8,576 (8.2%)*
Humerus	*14,820 (12.2%)*	*24,606 (11.5%)*	*8,600 (3.2%)*	*1,728 (1.6%)*
Hand	*3,809 (3.1%)*	*7,610 (3.5%)*	*20,940 (7.7%)*	*14,475 (13.8%)*
Clavicle	*2,678 (2.2%)*	*399 (0.2%)*	*2,403 (0.9%)*	*2,544 (2.4%)*
Scapula	*9 (0.0%)*	*9 (0.0%)*	*100 (0.0%)*	*126 (0.1%)*
Upper limb, unspecified	*9 (0.0%)*	*58 (0.0%)*	*95 (0.0%)*	*22(0.0%)*
Shoulder, unspecified	*32 (0.0%)*	*16 (0.0%)*	*53 (0.0%)*	*37(0.0%)*
**Lower limb**	17,292 (14.2%)	12,497 (5.8%)	37,622 (13.8%)	18,326 (17.4%)
Tibia/Fibula	*7,670 (6.3%)*	*8,088 (3.8%)*	*28,470 (0.4%)*	*12,501 (11.9%)*
Femur	*8,382 (6.9%)*	*2,804 (1.3%)*	*2,991 (1.1%)*	*1,856 (1.8%)*
Foot (not calcaneum or talus)	*613 (0.5%)*	*875 (0.4%)*	*2,418 (0.9%)*	*1,630 (1.6%)*
Hip	*522 (0.4%)*	*316 (0.1%)*	*1,244 (0.5%)*	*346 (0.3%)*
Pelvis	*56 (0.0%)*	*143 (0.1%)*	*936 (0.0%)*	*848 (0.8%)*
Patella	*13 (0.0%)*	*166 (0.1%)*	*1,126 (6.3%)*	*654 (0.6%)*
Talus	*10 (0.0%)*	*42 (0.0%)*	*239 (0.1%)*	*275 (0.3%)*
Calcaneum	*26 (0.0%)*	*63 (0.0%)*	*198 (0.1%)*	*216 (0.2%)*
**Spine**	112 (0.1%)	249 (0.1%)	1,451 (0.0%)	2,730 (2.6%)
Thoracic spine	*18 (0.0%)*	*106 (0.0%)*	*599 (0.2%)*	*911 (0.9%)*
Lumbar spine	*11 (0.0%)*	*69 (0.0%)*	*479 (0.2%)*	*1,058 (1.0%)*
Cervical spine	*80 (0.1%)*	*56 (0.0%)*	*294 (0.1%)*	*555 (0.5%)*
Sacral spine	*3 (0.0%)*	*18 (0.0%)*	*79 (0.0%)*	*206 (0.2%)*
**Skull and facial bones**	6,970 (5.7%)	2,025 (0.9%)	4,303 (1.6%)	7,641 (7.3%)
**Chest**	256 (0.2%)	64 (0.0%)	220 (0.1%)	354 (0.3%)

Confidential: For Review Only

Figure 1 Trends of fracture incidence for upper limb, lower limb, skull and facial fractures and spinal fractures between 2012-2019. Data presented separated by the age groups of children with trends and 95% confidence intervals calculated using a linear regression model.

Confidential: For Review Only

Table 3 Mean incidence of fractures per 100,000 person years (PY) admitted to hospitals in England between 2012-2019 according to age of child.

**Body region**	**Preschool Age 0-4**	**Younger children Age 5-9**	**Older children Age 10-15**	**Adolescents Age 16-18**
**Upper limb**	146.9	348.3	319.9	180.4
Forearm	*68.2*	*226.9*	*211.6*	*56.3*
Humerus	*54.6*	*91.3*	*28.9*	*11.3*
Hand	*14.0*	*28.3*	*70.5*	*94.9*
Clavicle	*9.9*	*1.5*	*8.1*	*16.7*
Scapula	*<0.1*	*<0.1*	*0/3*	*0.8*
Upper limb, unspecified	*<0.1*	*0.2*	*0.3*	*0.1*
Shoulder, unspecified	*0.1*	*0.1*	*0.2*	*0.2*
**Lower limb**	63.7	46.7	126.5	120.3
Tibia/Fibula	*28.2*	*30.2*	*95.7*	*82.1*
Femur	*30.9*	*10.4*	*10.1*	*12.2*
Foot (not calcaneum or talus)	*2.3*	*3.3*	*8.1*	*10.7*
Hip	*1.9*	*1.2*	*4.2*	*2.3*
Pelvis	*0.2*	*0.5*	*3.1*	*5.6*
Patella	*<0.1*	*0.6*	*3.8*	*4.3*
Talus	*<0.1*	*0.2*	*0.8*	*1.8*
Calcaneum	*0.1*	*0.2*	*0.7*	*1.4*
**Spine**	0.4	0.9	4.9	17.9
Thoracic spine	*0.1*	*0.4*	*2.0*	*6.0*
Lumbar spine	*<0.1*	*0.3*	*1.6*	*7.0*
Cervical spine	*0.3*	*0.2*	*1.0*	*16.7*
Sacral spine	*<0.1*	*0.1*	*0.3*	*1.4*
**Skull and facial bones**	25.7	7.5	14.5	50.1
**Chest**	0.9	0.2	0.7	2.3

Figure 2 Trends of fracture interventions for limb open or closed reductions, skull and facial fractures and spinal fractures between 2012-2019. Data presented separated by the age groups of children with trends and 95% confidence intervals calculated using a linear regression model.

Confidential: For Review Only

1. Landin LA. Fracture patterns in children. Analysis of 8,682 fractures with special reference to incidence, etiology and secular changes in a Swedish urban population 1950-1979. *Acta orthopaedica Scandinavica Supplementum* 1983;202:1-109. [published Online First: 1983/01/01]
2. Tiderius CJ, Landin L, Düppe H. Decreasing incidence of fractures in children: an epidemiological analysis of 1,673 fractures in Malmö, Sweden, 1993-1994. *Acta Orthop Scand* 1999;70(6):622-6. doi: 10.3109/17453679908997853 [published Online First: 2000/02/09]
3. Kang MS, Kim HS. Characteristics and trends of traumatic injuries in children visiting emergency departments in South Korea: A retrospective serial cross-sectional study using both nationwide-sample and single-institutional data. *PLoS One* 2019;14(8):e0220798. doi: 10.1371/journal.pone.0220798 [published Online First: 2019/08/10]
4. Bergman E, Lempesis V, Nilsson J-Å, et al. Time trends in pediatric fractures in a Swedish city from 1950 to 2016. *Acta Orthopaedica* 2020;91(5):598-604. doi: 10.1080/17453674.2020.1783484
5. Orces CH, Orces J. Trends in the U.S. Childhood Emergency Department Visits for Fall-Related Fractures, 2001-2015. *Cureus* 2020;12(11):e11629. doi: 10.7759/cureus.11629 [published Online First: 2020/12/31]
6. Ding R, McCarthy ML, Houseknecht E, et al. The health-related quality of life of children with an extremity fracture: a one-year follow-up study. *J Pediatr Orthop* 2006;26(2):157-63. doi: 10.1097/01.bpo.0000218521.98244.7e [published Online First: 2006/03/25]
7. Kendrick D, Vinogradova Y, Coupland C, et al. Recovery from injury: the UK burden of injury multicentre longitudinal study. *Injury prevention* 2013;19(6):370-81.
8. Schneeberg A, Ishikawa T, Kruse S, et al. A longitudinal study on quality of life after injury in children. *Health and quality of life outcomes* 2016;14(1):120.
9. FANZCA M. Distress at induction of anaesthesia in children. A survey of incidence, associated factors and recovery characteristics. *Pediatric Anesthesia* 1998;8(5):383-92.
10. Bentley J. Distress in children attending A&E. *Emergency nurse* 2004;12(4)
11. Murphy T, Akehurst H, Mutimer J. Impact of the 2020 COVID-19 pandemic on the workload of the orthopaedic service in a busy UK district general hospital. *Injury* 2020;51(10):2142-47.
12. Sugand K, Park C, Morgan C, et al. Impact of the COVID-19 pandemic on paediatric orthopaedic trauma workload in central London: a multi-centre longitudinal observational study over the "golden weeks". *Acta Orthop* 2020;91(6):633-38. doi: 10.1080/17453674.2020.1807092 [published Online First: 2020/08/25]
13. Digital N. OPCS Classification of Interventions and Procedures 2021 [Available from: https://datadictionary.nhs.uk/supporting_information/opcs_classification_of_interventions_and_procedures.html accessed 30/4/2021.
14. Organization WH. ICD-10 : international statistical classification of diseases and related health problems : tenth revision. 2nd ed. Geneva: World Health Organization. , 2004.
15. Murphy A. Reported road casualties in Great Britain: 2019 annual report. London: Department for Transport, 2020.
16. Transport Df. Think! 2021 [Available from: https://www.think.gov.uk/ accessed 30/4/2021.
17. Allen G, Kirk-Wade E. Knife crime in England and Wales. Briefing Paper. London: House of Commons Library, 2020.
18. Kurien T, Price K, Pearson RG, et al. Manipulation and reduction of paediatric fractures of the distal radius and forearm using intranasal diamorphine and 50% oxygen and nitrous oxide in the emergency department: a 2.5-year study. *The bone & joint journal* 2016;98(1):131-36.
19. Marson BA, Ng JW, Craxford S, et al. Treatment of completely displaced distal radial fractures with a straight plaster or manipulation under anaesthesia. *The Bone & Joint Journal* 2021:1-6.
20. Patel D, Talbot C, Luo W, et al. The use of esketamine sedation in the emergency department for manipulation of paediatric forearm fractures: A 5 year study. *Injury* 2021

Confidential: For Review Only

21. Yap RY, Sultan S, Ahmad H, et al. Early Opiate Analgesic Requirements Following Nonsurgically Managed Tibial Fractures in Children. *Journal of Pediatric Orthopaedics* 2021;41(8):467-71.
22. Marson BA, Ng J, Myint Y, et al. Management of 'low-risk' ankle fractures in children: a systematic review. *Ann R Coll Surg Engl* 2019;101(8):539-45. doi: 10.1308/rcsann.2019.0027 [published Online First: 2019/03/12]
23. Faris M, Lystad RP, Harris I, et al. Fracture-related hospitalisations and readmissions of Australian children ≤16 years: A 10-year population-based cohort study. *Injury* 2020;51(10):2172-78. doi: 10.1016/j.injury.2020.07.032 [published Online First: 2020/07/28]
24. Moon RJ, Harvey NC, Curtis EM, et al. Ethnic and geographic variations in the epidemiology of childhood fractures in the United Kingdom. *Bone* 2016;85:9-14. doi: 10.1016/j.bone.2016.01.015 [published Online First: 2016/01/24]
25. Orton E, Kendrick D, West J, et al. Persistence of health inequalities in childhood injury in the UK: a population-based cohort study of children under 5. *PLoS One* 2014;9(10):e111631. doi: 10.1371/journal.pone.0111631 [published Online First: 2014/10/28]
26. Rosenfeld EH, Johnson B, Wesson DE, et al. Understanding non-accidental trauma in the United States: A national trauma databank study. *Journal of Pediatric Surgery* 2020;55(4):693-97. doi: https://doi.org/10.1016/j.jpedsurg.2019.03.024
27. Thompson LW, Bass K, Agyei J, et al. Rising Incidence of Non-Accidental Trauma in Infants: A Call to Revisit Prevention Strategies. *Pediatrics* 2018;142:749-49. doi: 10.1542/peds.142.1_MeetingAbstract.749
28. Bram JT, Johnson MA, Magee LC, et al. Where have all the fractures gone? The epidemiology of pediatric fractures during the COVID-19 pandemic. *Journal of Pediatric Orthopaedics* 2020;40(8):373-79.
29. Nabian MH, Vosoughi F, Najafi F, et al. Epidemiological pattern of pediatric trauma in COVID-19 outbreak: Data from a tertiary trauma center in Iran. *Injury* 2020;51(12):2811-15. doi: 10.1016/j.injury.2020.09.015 [published Online First: 2020/09/23]
30. Pines JM, Zocchi MS, Black BS, et al. Characterizing pediatric emergency department visits during the COVID-19 pandemic. *The American journal of emergency medicine* 2021;41:201-04.
31. Ibrahim Y, Huq S, Shanmuganathan K, et al. Trampolines injuries are bouncing back: The effect of the COVID-19 UK lockdown on the paediatric trauma burden. *Bone & Joint Open* 2021;2(2):86.
32. Judge A, Murphy R, Maxwell R, et al. Temporal trends and geographical variation in the use of subacromial decompression and rotator cuff repair of the shoulder in England. *The bone & joint journal* 2014;96(1):70-74.
33. Manley OW, Wormald JC, Furniss D. The changing shape of hand trauma: an analysis of Hospital Episode Statistics in England. *Journal of hand surgery (European volume)* 2019;44(5):532-36.
34. Digital N. HES Data Quality Statement 2020 [Available from: https://digital.nhs.uk/data-and-information/publications/statistical/hospital-admitted-patient-care-activity/2019-20/data-quality-statement accessed 30/4/2021.

Confidential: For Review Only

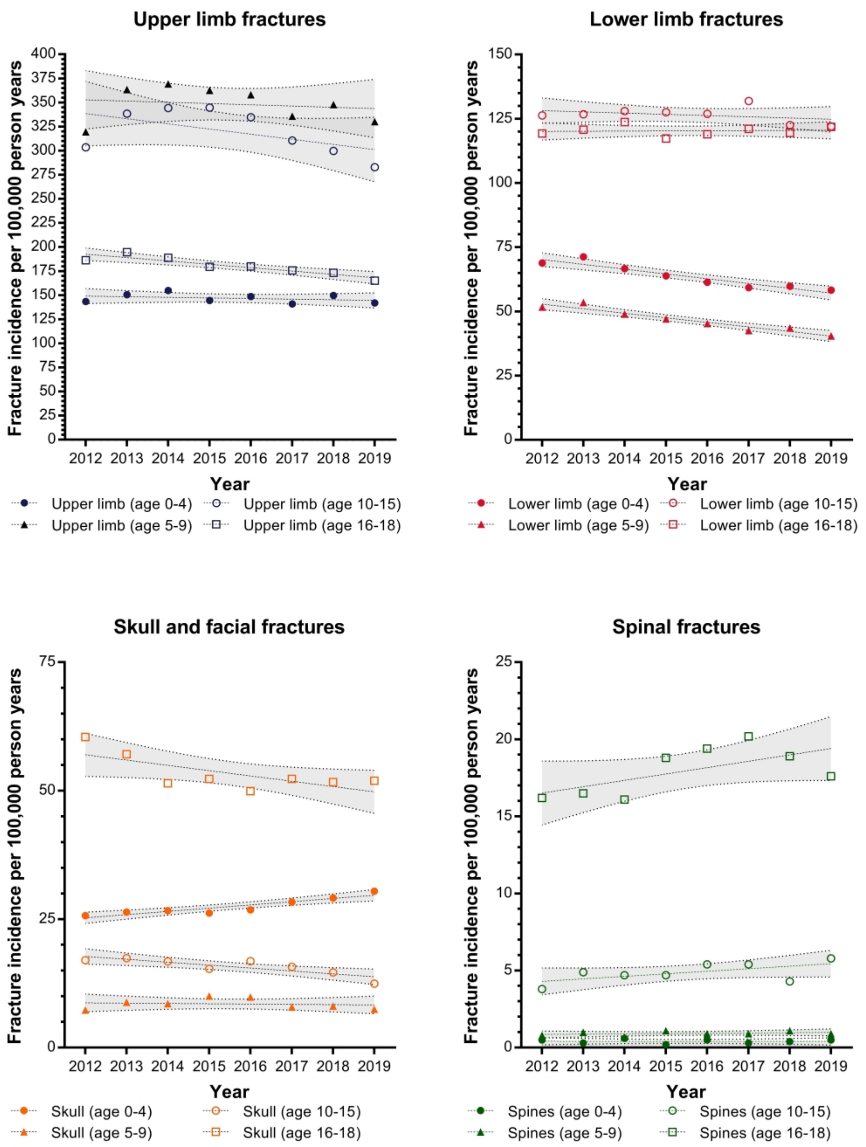

200x253mm (600 x 600 DPI)

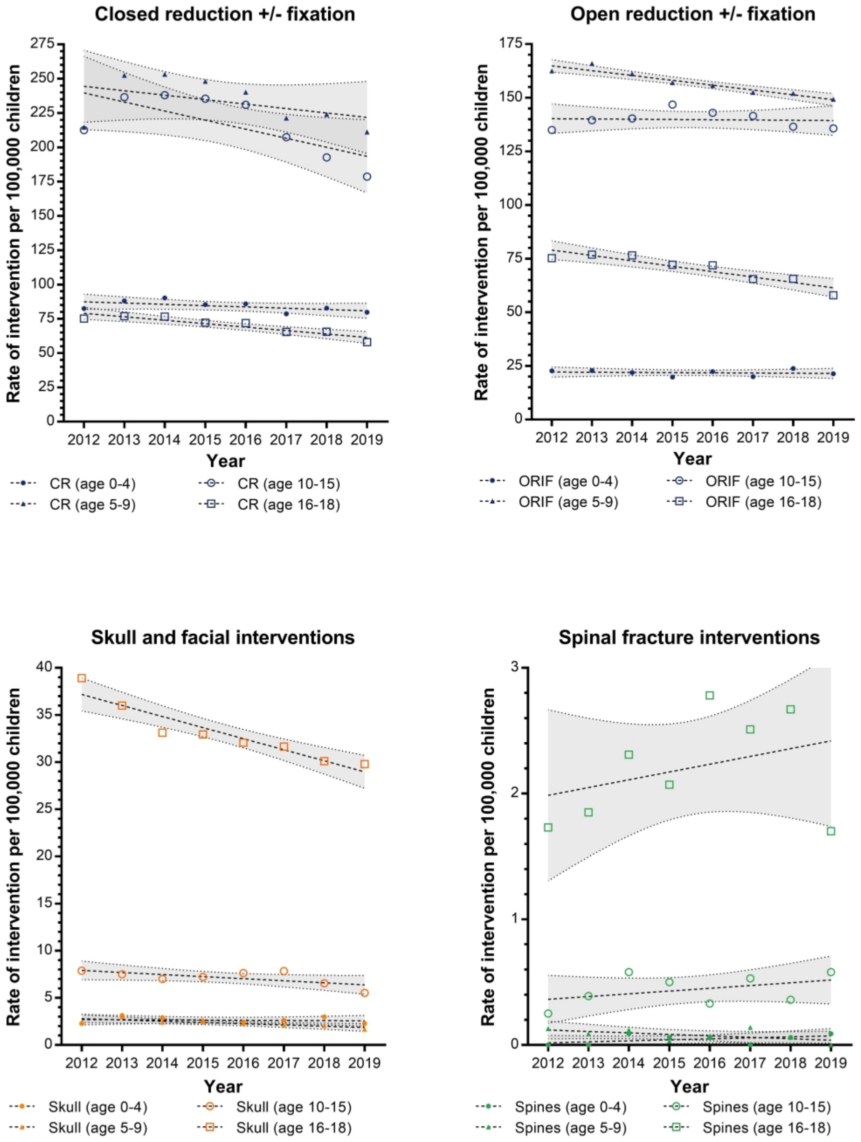

195x247mm (600 x 600 DPI)

List of included codes

code	Name	Mapped to body area
S12.0	Fracture of first cervical vertebra	C-spine
S12.1	Fracture of second cervical vertebra	C-spine
S12.2	Fracture of other specified cervical vertebra	C-spine
S12.8	Fracture of other parts of neck	C-spine
S12.9	Fracture of neck, part unspecified	C-spine
S62.0	Fracture of navicular [scaphoid] bone of han	carpus
S62.1	Fracture of other carpal bone(s)	carpus
P13.4	Fracture of clavicle due to birth injury	clavicle
S42.0	Fracture of clavicle	clavicle
S72.3	Fracture of shaft of femur	femur
S72.4	Fracture of lower end of femur	femur
S72.8	Fractures of other parts of femur	femur
S72.9	Fracture of femur, part unspecified	femur
S92.9	Fracture of foot, unspecified	foot
S52.0	Fracture of upper end of ulna	Forearm
S52.1	Fracture of upper end of radius	Forearm
S52.2	Fracture of shaft of ulna	Forearm
S52.3	Fracture of shaft of radius	Forearm
S52.4	Fracture of shafts of both ulna and radius	Forearm
S52.5	Fracture of lower end of radius	Forearm
S52.6	Fracture of lower end of both ulna and radiu	Forearm
S52.8	Fracture of other parts of forearm	Forearm
S52.9	Fracture of forearm, part unspecified	Forearm
S62.5	Fracture of thumb	hand
S62.6	Fracture of other finger	hand
S62.8	Fracture of other and unspecified parts of wr	hand
S92.0	Fracture of calcaneus	hindfoot
S92.1	Fracture of talus	hindfoot
S72.0	Fracture of neck of femur	hip
S72.1	Pertrochanteric fracture	hip
S72.2	Subtrochanteric fracture	hip
S42.2	Fracture of upper end of humerus	humerus
S42.3	Fracture of shaft of humerus	humerus
S42.4	Fracture of lower end of humerus	humerus
S32.0	Fracture of lumbar vertebra	L-spine
S82.1	Fracture of upper end of tibia	lower leg
S82.2	Fracture of shaft of tibia	lower leg
S82.3	Fracture of lower end of tibia	lower leg
S82.4	Fracture of fibula alone	lower leg
S82.5	Fracture of medial malleolus	lower leg
S82.6	Fracture of lateral malleolus	lower leg
S82.8	Fractures of other parts of lower leg	lower leg
S82.9	Fracture of lower leg, part unspecified	lower leg
S62.2	Fracture of first metacarpal bone	metacarp
S62.3	Fracture of other metacarpal bone	metacarp
S82.0	Fracture of patella	patella
S32.1	Fracture of sacrum	pelvis
S32.2	Fracture of coccyx	pelvis
S32.3	Fracture of ilium	pelvis
S32.4	Fracture of acetabulum	pelvis
S32.5	Fracture of pubis	pelvis
S32.8	Fracture of other and unspecified parts of lu	pelvis
S42.1	Fracture of scapula	scapula
P13.0	Fracture of skull due to birth injury	skull
S02.0	Fracture of vault of skull	skull
S02.1	Fracture of base of skull	skull
S02.3	Fracture of orbital floor	skull
S02.4	Fracture of malar and maxillary bones	skull
S02.6	Fracture of mandible	skull
S02.8	Fractures of other skull and facial bones	skull
S02.9	Fracture of skull and facial bones, part unsp	skull

Confidential: For Review Only

T08.X	Fracture of spine, level unspecified	spine
S22.0	Fracture of thoracic vertebra	t0spine
S92.2	Fracture of other tarsal bone(s)	tarsal
S92.3	Fracture of metatarsal bone	tarsal
S22.2	Fracture of sternum	thorax
S22.3	Fracture of rib	thorax
S22.8	Fracture of other parts of bony thorax	thorax
S22.9	Fracture of bony thorax, part unspecified	thorax
S92.4	Fracture of great toe	toe
S92.5	Fracture of other toe	toe

Confidential: For Review Only
